# Machine Learning-Guided Virtual Screening Identifies a Novel MELK-Targeted Antiproliferative Lead Compound with G2/M Arrest Activity in Triple-Negative Breast Cancer and Lung Adenocarcinoma Cells

**DOI:** 10.3390/cancers18203336

**Published:** 2026-10-15

**Authors:** Wanxin Cao, Bin Jiang, Xiangyin Chi, Wan Li, Hong Yang, Xiaoxue Li, Yuxuan Zhao, Xu Zhang, Rui Meng, Yanyu Cang, Guanhua Du, Jinhua Wang

**Affiliations:** 1 Beijing Key Laboratory of Innovative Drug Discovery and Polymorphic Druggability Research for Cerebrovascular Diseases, Institute of Materia Medica, Chinese Academy of Medical Sciences & Peking Union Medical College, Beijing 100050, China; caowanxin@imm.ac.cn (W.C.); jiangbin@imm.ac.cn (B.J.); chixiangyin@imm.ac.cn (X.C.); liwan@imm.ac.cn (W.L.); yang.hong@imm.ac.cn (H.Y.); zhangxu2024@imm.ac.cn (X.Z.); cangyanyu@imm.ac.cn (Y.C.); dugh@imm.ac.cn (G.D.); 2 School of Clinical Pharmacy, Shenyang Pharmaceutical University, Shenyang 117004, China; xue07121001@163.com; 3 School of Pharmacy, Henan University, Kaifeng 475004, China; zyx05032024@163.com; 4 Modern Research Center for Traditional Chinese Medicine, Shanxi University, Taiyuan 030006, China; 202424324025@email.sxu.edu.cn

**Keywords:** MELK, virtual screening, machine learning, triple-negative breast cancer, lung adenocarcinoma, CETSA

## Abstract

**Background:** Maternal embryonic leucine zipper kinase (MELK) is a cell cycle-regulated serine/threonine kinase frequently overexpressed in human malignancies, and represents a promising anticancer drug target. Discovery of structurally novel, highly selective MELK inhibitors remains an unmet need. **Methods:** In the study, we implemented an integrated computational pipeline combining four machine learning regressors (Random Forest, Gradient Boosting, XGBoost, LightGBM), hierarchical Glide docking (HTVS to SP to XP), MM/GBSA rescoring, ADMET prediction, and 100 ns molecular dynamics simulations to screen a ~15.58 million-compound commercial library, yielding 12 prioritized candidates. Three commercially available compounds (CPD4, CPD6, CPD10) were evaluated in an antiproliferative panel. **Results:** CPD6 was identified as the most active hit, with IC_50_ values of 1.44 μM in MDA-MB-468 and 1.66 μM in H1299, modestly exceeding the reference compound MELK-8a under the same assay conditions (2.48 and 2.51 μM, respectively). Cellular thermal shift assay in cell lysates provided biochemical evidence of MELK engagement, and 100 ns molecular dynamics simulations supported a computationally predicted binding mode in the MELK kinase domain. Additional substrate phosphorylation and MELK overexpression rescue assays further supported MELK-dependent inhibitory activity. Colony formation, EdU incorporation, and cell cycle analyses demonstrated dose-dependent antiproliferative effects and G2/M arrest, with concomitant downregulation of CDC25C, CDK1, and Cyclin B1. **Discussion:** CPD6 is a structurally novel MELK lead distinct from reported inhibitors. While our data support MELK target engagement, kinome selectivity profiling remains required, and this ML pipeline demonstrates the utility of AI-driven large-scale anticancer lead screening. **Conclusions:** Using the integrated machine-learning virtual screening pipeline established in this work, we identified CPD6 as a structurally novel MELK-targeted lead that engages MELK, induces G2/M arrest, and exerts low-micromolar antiproliferative activity in triple-negative breast cancer and lung adenocarcinoma models.

## Simple Summary

Maternal embryonic leucine zipper kinase (MELK) is a promising anticancer target, but the discovery of novel MELK inhibitors remains challenging due to the inefficiency of conventional high-throughput screening approaches. Here, we established an integrated machine learning-guided virtual screening workflow combining hierarchical docking, MM/GBSA rescoring, ADMET prediction, and molecular dynamics simulations to prioritize 12 candidates from approximately 15.58 million compounds. Among three commercially available compounds tested, CPD6 emerged as a structurally novel lead with low-micromolar antiproliferative activity against triple-negative breast cancer (MDA-MB-468) and lung adenocarcinoma (H1299) cells, outperforming the reference inhibitor MELK-8a. Cellular thermal shift assay in cell lysates provided biochemical evidence of MELK engagement, and functional assays demonstrated that CPD6 suppresses colony formation, inhibits DNA synthesis, and induces G2/M arrest via downregulation of the CDC25C-CDK1-Cyclin B1 axis. These findings support CPD6 as a cell-active lead for further optimization and validate the utility of this computational pipeline in anticancer drug discovery.

## 1. Introduction

Despite significant advances in targeted therapies, cancer remains a major cause of mortality worldwide, with the development of safe and effective drugs particularly constrained in the context of chemo- and radio-resistance [1], emphasizing the continued necessity for the identification of novel, mechanism-based targets and their therapeutic agents.

Maternal Embryonic Leucine Zipper Kinase (MELK), a cell cycle-dependent kinase of the Snf1/AMPK family [2], plays critical roles in regulating cell cycle progression [3], proliferation [4], stemness maintenance [5], and post-translational modifications [6]. Clinical cohort studies and in vitro/in vivo experiments have shown that MELK is overexpressed in multiple cancers, including lung adenocarcinoma [3], breast cancer [7], hepatocellular carcinoma [8] and cervical cancer [4], and its high expression is closely associated with multidrug resistance [9] and radio-resistance [7]. These findings suggest that MELK represents a potential target for therapeutic strategies aimed at enhancing chemo- and radiotherapy efficacy.

The MELK inhibitor OTSSP167 has been widely used to interrogate MELK function [10], but kinobead profiling showed that it binds with high affinity to more than 100 kinases, placing it among the least selective MELK inhibitors reported to date [11]. McDonald further showed that OTSSP167 inhibited at least 12 kinases more potently than MELK, indicating that its antitumor activity likely arises from off-target kinase inhibition rather than MELK blockade [12].

Over the past few years, several highly selective MELK inhibitors have been developed, such as MELK-8a, MELK-T1, MELK-IN-1 and HTH-01-091 [1]. MELK-8a shows exceptional selectivity and target fidelity at both enzymatic and cellular levels [13]. These properties support further studies of MELK and the development of MELK-targeted therapeutics. Conventional drug screening and optimization approaches remain limited by low efficiency, high costs, and long experimental validation cycles. These limitations restrict the discovery of novel, highly selective MELK inhibitors. The application of artificial intelligence has accelerated the development of drug discovery. Machine learning–based virtual screening has become a powerful method to improve the efficiency and accuracy of compound selection.

To overcome these bottlenecks in MELK inhibitor discovery, we developed an integrated machine learning-driven virtual screening workflow to identify structurally novel MELK-targeted small molecules. We first curated a high-quality dataset of experimentally validated MELK inhibitors to train and compare multiple regression models for bioactivity prediction, which was combined with hierarchical molecular docking, MM/GBSA binding free energy rescoring, in silico ADMET profiling, and 100 ns molecular dynamics simulations to prioritize candidates from a 15.58 million commercially available screening library. A subset of top-ranked, commercially purchasable compounds was then subjected to in vitro antiproliferative evaluation across MELK-relevant cancer cell lines, followed by preliminary cellular target engagement and functional mechanistic assays. This study was designed to identify chemically distinct, cell-active lead compounds for future MELK-focused hit-to-lead optimization, and to establish a streamlined, cost-effective computational-to-experimental pipeline for anticancer lead discovery that can be generalized to other oncology targets.

## 2. Materials and Methods

### 2.1. Data Collection

A high-quality, multi-source dataset was curated by integrating 1093 experimentally validated MELK inhibitors from the ChEMBL database (CHEMBL4578 [14]), 12 peer-reviewed publications [15,16,17,18,19,20,21,22,23,24,25,26], and 3 granted patent families (CN104968345B, US10544138B2, US20210317107A1). To mitigate variability derived from distinct experimental assay formats, only compounds tested under consistent or comparable experimental systems were retained for unified analysis. Duplicate entries were standardized using SMILES as the unique identifier: for compounds with repeated IC_50_ measurements, values with a deviation ≤ 0.01 mM were averaged to generate a unified pIC_50_, while inconsistent entries with larger deviations were removed. All data were unified, standardized, deduplicated, and converted to pIC_50_ for consistent model training. The resulting dataset covers a wide range of inhibitory activities, spanning pIC_50_ values from 4.0 to 9.0, which corresponds to IC_50_ values from 100 μM to 1 nM, encompassing weak, moderate, and highly active compounds. Despite the modest dataset size, all activity values are experimentally derived and uniformly annotated, ensuring high reliability for model construction.

### 2.2. Molecular Descriptor Calculation and Machine Learning Model Construction

To comprehensively capture molecular structure and property information, multi-modal molecular descriptors were calculated using the RDKit library. The feature set included 2D physicochemical descriptors (molecular weight, LogP, topological polar surface area, HBA/HBD, rotatable bonds), molecular fingerprints (Morgan fingerprints, RDKit fingerprints, MACCS 166-bit keys), topological indices (Balaban J, Zagreb indices), ring statistics (saturated, aromatic, aliphatic rings), as well as heteroatom count, valence electrons, radical electrons, and QED drug-likeness score.

We randomly split the dataset of 1093 molecules into an 80:20 ratio using scikit-learn’s ‘train_test_split’ function, with 80% for training and 20% for testing. To evaluate the ability of different regression algorithms to predict pIC_50_ values, we compared multiple classical models including LightGBM, GBR, Random Forest, XGBoost, Ridge, SVR, KNN, PLS, MLP, DTR, and ElasticNet. All models in this study were implemented using default hyperparameters from the scikit-learn library, with only random_state fixed to ensure full reproducibility. No additional hyperparameter optimization was performed. This ensures transparency and facilitates complete reproducibility of all results. Model performance was comprehensively evaluated using four standard regression metrics: the coefficient of determination (*R*^2^), mean squared error (MSE), root-mean-square error (RMSE), and mean absolute error (MAE). To validate robustness and avoid overfitting, 10-fold cross-validation was performed on the training set, with performance reported as mean ± standard deviation.

To comprehensively evaluate the predictive performance of regression models for pIC_50_ estimation, four widely adopted metrics were employed, with consistent notation defined as follows: *y_i_* denotes the experimentally measured true pIC_50_ value for the *i*-th sample, ${\hat{y}}_{i}$ denotes the model-predicted pIC_50_ value for the *i*-th sample, $\bar{y}$ denotes the mean of all true pIC_50_ values, and *n* denotes the total number of samples.

The coefficient of determination (*R*^2^) evaluates the goodness of fit of a model and is defined as: $R^{2}=1-\frac{\sum_{i=1}^{n}\;{\left(y_{i}-{\hat{y}}_{i}\right)}^{2}}{\sum_{i=1}^{n}\;{\left(y_{i}-\bar{y}\right)}^{2}}$. *R*^2^ ranges from 0 to 1, where a higher value indicates a better model fit: an *R*^2^ = 1 represents perfect prediction, while *R*^2^ = 0 indicates the model explains no variance in the target variable.

The root mean squared error (RMSE) is the square root of MSE, converting errors back to the original unit of the target variable (pIC_50_ in this study) for intuitive interpretability: $\mathrm{RMSE}=\sqrt{\frac{1}{n}\Sigma_{i=1}^{n}{\left({\hat{y}}_{i}-y_{i}\right)}^{2}}$. RMSE quantifies the average magnitude of prediction errors, with a lower value indicating higher predictive reliability. Unlike MSE, RMSE avoids artificial amplification of large errors from squaring, making it more suitable for directly reflecting actual prediction deviation.

The mean absolute error (MAE) calculates the average absolute difference between predicted and true values, focusing on absolute error magnitude without amplifying extreme outlier impact: $\mathrm{MAE}=\frac{1}{n}\Sigma_{i=1}^{n}\left|{\hat{y}}_{i}-y_{i}\right|$. A lower MAE indicates more stable predictive performance, less sensitive to outliers.

By jointly analyzing *R*^2^, RMSE, and MAE, we systematically evaluated the performance of different regression models and selected the optimal model for subsequent virtual screening.

### 2.3. Database Preparation

Compounds were collected from ZINC (Drug-like and Lead-like) [27], Chemdiv (D001), and MCE (HY-L901P) databases. After duplicate removal, a final non-redundant library containing 15,576,893 compounds was obtained for virtual screening.

### 2.4. Virtual Screening

Four machine learning models including Random Forest, GBR, LightGBM, and XGBoost were used to predict the pIC_50_ values of compounds. Compounds showing pIC_50_ values above 7 in at least three models were selected as potential candidates. We then performed molecular docking and binding affinity analysis to assess their interactions with the MELK protein.

### 2.5. Applicability Domain Analysis

To evaluate the applicability domain and ensure the reliability of the predictive model for the virtual screening library, t-distributed stochastic neighbor embedding (t-SNE) visualization was performed. The chemical space of the 15.58 million virtual screening library was characterized by 1024-bit Morgan fingerprints. For computational efficiency, 150,000 molecules were randomly sampled from the entire library to generate a representative chemical space. The t-SNE algorithm was employed to reduce the high-dimensional fingerprint data into two dimensions with a perplexity of 80. The distributions of the training set molecules and the sampled virtual library molecules were visualized on the same map to assess whether the training set was well embedded within the library space, thereby verifying the model’s predictive scope.

### 2.6. Molecular Docking

Before docking, the 3D structures of the molecules were prepared using the LigPrep module in the Schrödinger Suite 12.9. The procedure included energy minimization under the OPLS4 force field, protonation state prediction with the Epik method at pH 7.4 ± 0.5, removal of salts or ions, and generation of tautomers. Chiral centers were handled using the ‘Retain specified chiralities’ option. The crystal structure of the MELK protein (PDB ID: 5K00) retrieved from the PDB database (https://www.rcsb.org/) [28] was used as the basis for virtual screening.

The protein structure was prepared using the Protein Preparation Wizard in Maestro 12.9, which included assignment of bond orders, addition and optimization of hydrogen atoms, prediction of protonation states at pH 7.4, and removal of crystallographic water molecules. The structure was then subjected to restrained energy minimization under the OPLS4 force field to optimize its conformation and reduce geometric deviations. A receptor grid was generated using the Receptor Grid Generation module by first excluding all ligands to define the receptor, centering the docking box on the geometric center of the co-crystal ligand, and setting the van der Waals scaling factor and partial charge cutoff to 1.0 and 0.25, respectively, to optimize nonpolar interactions. This prepared grid was used for small-molecule docking, which was performed with the Ligand Docking module using sequential HTVS, SP, and XP protocols, retaining only the top 20% of compounds based on docking scores at each stage for subsequent screening. The docking poses were visualized using PyMOL (version 4.6), which allowed clear depiction of ligand–protein interactions including hydrogen bonds and hydrophobic contacts.

### 2.7. MM/GBSA Binding Free Energy Calculation

The binding free energy (ΔG_bind) of ligand–receptor complexes was calculated with the Prime MM/GBSA module using the VSGB solvation model and OPLS4 force field. Complexes were extracted from Maestro Pose Viewer docking outputs, energy-minimized, and rescored; more negative ΔG_bind values indicate more favorable computed pose energetics. A pragmatic enrichment cutoff (ΔG_bind ≤ −50 kcal/mol and ΔG_bind more favorable than the re-docked MELK-8a reference pose) was then applied to narrow candidates for visual inspection and purchase. This cutoff is used strictly as a relative ranking feature within the screening workflow and is not interpreted as evidence of superior experimental binding affinity relative to MELK-8a, given the approximate nature of MM/GBSA scoring and its uncertainty of several kcal/mol in absolute computed values.

### 2.8. ADMET Prediction

The ADMET properties of the candidate MELK-targeted compounds were predicted using the ADMETLab 3.0 [29] online platform, a widely used and well-validated online platform extensively adopted in computational drug discovery [30,31,32,33]. The SMILES strings of the compounds were submitted to the platform, which automatically generated predictions of their ADMET profiles.

### 2.9. Molecular Dynamics Simulation

Molecular dynamics simulations were performed using the GROMACS 2022 software package [34]. Force field parameters for the small-molecule ligands were generated via the CGenFF [35] web server. Protein–ligand complexes were modeled under the CHARMM36m force field and solvated using the TIP3P water model. Appropriate amounts of Na^+^ or Cl^−^ ions were added to neutralize the system. Energy minimization was carried out using the steepest descent algorithm until the maximum force fell below 1000 kJ/mol/nm. The system then underwent 100 ps of NVT equilibration followed by 100 ps of NPT (Constant Number, Pressure, Temperature ensemble) equilibration. During NVT (Constant Number, Volume, Temperature ensemble) equilibration, the temperature was maintained at 298.15 K using the V-rescale thermostat. During NPT equilibration, the pressure was set to 1 bar using the Parrinello–Rahman barostat. Production MD simulations were conducted for 100 ns with a time step of 2 fs. Coordinates and energy data were saved every 10 ps for subsequent analysis. The RMSD (Root Mean Square Deviation) of the protein backbone, ligand, and the complex was calculated using GROMACS built-in tools to assess the stability of the system throughout the simulation. Structural snapshots were visualized using VMD 2.0 software.

### 2.10. Cell Culture

Six human cancer cell lines and one non-cancerous epithelial cell line were used in this study: the triple-negative breast cancer lines MDA-MB-231 and MDA-MB-468, the non-small-cell lung cancer lines H1299 and H1975, the hepatocellular carcinoma line HepG2, the esophageal squamous-cell carcinoma line KYSE30, and the non-cancerous human bronchial epithelial line BEAS-2B. All lines were obtained from ATCC. MDA-MB-231, H1975, HepG2, and KYSE30 were used in the initial single-concentration screen; MDA-MB-468 and H1299 were introduced in the follow-up dose–response and mechanistic studies on the basis of literature-documented MELK expression and their use as model systems in prior MELK functional studies [3,8,36,37]. BEAS-2B was used to assess non-specific general cytotoxicity and calculate the preliminary selectivity window of CPD6.

The MDA-MB-231, MDA-MB-468, H1299, HepG2, and BEAS-2B cell lines were cultured in DMEM medium (PM150210, Procell, Wuhan, China) supplemented with 10% fetal bovine serum (FBS, 164210-50, Procell, Wuhan, China) and 1% penicillin-streptomycin (P1400, Solarbio, Beijing, China). The H1975 and KYSE30 cell lines were cultured in RPMI-1640 medium (PM150110, Procell, Wuhan, China) supplemented with 10% fetal bovine serum (FBS, 164210-50, Procell, Wuhan, China) and 1% penicillin-streptomycin (P1400, Solarbio, Beijing, China).

### 2.11. Compound Sources and In Vitro Proliferation Assay

Compounds CPD4, CPD6, and CPD10 were purchased from ChemBridge, and the reference inhibitor MELK-8a was obtained from Yuanye Bio-Technology (Shanghai, China). All compounds were dissolved in dimethyl sulfoxide (DMSO; Merck KGaA, Darmstadt, Germany). Cell antiproliferative activity and viability were evaluated using the Cell Counting Kit-8 (CCK-8; Yeasen, Shanghai, China). Cell viability was assessed according to the manufacturer’s protocol, and absorbance was measured at 450 nm using a SpectraMax M5 microplate reader (Molecular Devices, Sunnyvale, CA, USA). All proliferation assays were performed with n = 3 replicates per condition.

### 2.12. Colony Formation Assay

MDA-MB-468 and H1299 cells were digested and counted, then seeded into 6-well plates at a density of approximately 700 cells per well. After the cells attached, the medium was replaced with 10% FBS DMEM containing CPD6 at final concentrations of 0, 0.75, 1.5, or 3 μmol/L, or 3 μmol/L MELK-8a. When visible colonies formed, the culture was terminated. Cells were washed gently three times with PBS at room temperature, fixed with 4% paraformaldehyde for 30 min, and stained with crystal violet for another 30 min. The plates were then rinsed with PBS, air-dried, and photographed.

### 2.13. EdU DNA Synthesis Assay

Cell proliferation was assessed using the BeyoClick™ EdU Cell Proliferation Kit with Alexa Fluor 555 (C0075S, Beyotime, Shanghai, China) according to the manufacturer’s instructions. MDA-MB-468 and H1299 cells were treated with CPD6 at final concentrations of 0, 0.75, 1.5, or 3 μmol/L, or with 3 μmol/L MELK-8a for 72 h. EdU solution was then added to each well at a final concentration of 10 μmol/L, followed by incubation for an additional 4 h. Cells were fixed with 4% paraformaldehyde for 15 min at room temperature and permeabilized with 0.3% Triton X-100 for 15 min. Subsequently, cells were stained with Click reaction solution for 30 min and with DAPI for 10 min at room temperature. The stained cells were imaged using a high-content imaging system (Cellomics ArrayScan VTI, Thermo Fisher Scientific, Carlsbad, CA, USA).

### 2.14. Flow Cytometric Analysis of Cell Cycle

MDA-MB-468 and H1299 cells cultured in 60 mm dishes were treated with CPD6 at final concentrations of 0, 0.75, 1.5, and 3 μmol/L, or with 3 μmol/L MELK-8a for 72 h. Cells were collected, washed with PBS, and fixed in 70% ethanol at 4 °C overnight. After fixation, cells were washed three times with PBS and centrifuged at 1500 rpm for 5 min at 4 °C. The supernatant was discarded, and the cell pellet was resuspended and incubated with propidium iodide (PI) staining solution for 30 min at room temperature. Cell cycle distribution was analyzed using a BD Accuri C6 Plus flow cytometer (BD Biosciences, San Diego, CA, USA), and data were processed with FlowJo_V10 software.

### 2.15. Western Blot

Cells were harvested and lysed in RIPA lysis buffer (C1053-100, Gene-Protein Link, Beijing, China) on ice. After being centrifuged, the supernatant was mixed with SDS-PAGE Loading Buffer (CW0027, CWBIO, Beijing, China) and an equivalent amount (20 μg) was separated by SDS-PAGE electrophoresis, transferred to polyvinylidene fluoride (PVDF) membranes (IPVH00010, Millipore, Darmstadt, Germany), sealed up in 5% skim milk (P1622, Applygen, Beijing, China), and then incubated with corresponding diluted primary antibodies at 4 °C overnight. The information on the antibodies used in this study is summarized in Table S1. Subsequently, the membranes were washed three times with TBST and incubated with a secondary antibody conjugated to horseradish peroxidase at room temperature for 2 h. Finally, the membranes were washed three times with TBST and incubated with SuperECL (36208ES60, Yeasen, Shanghai, China). The membranes were examined using a chemiluminescence photodocumentation system (Tanon, Beijing, China), photographed and quantified.

### 2.16. Cellular Thermal Shift Assay (CETSA)

MDA-MB-468 and H1299 cells were seeded in 10 cm dishes and digested with 0.25% trypsin when the cell confluence reached 80%. The cells were collected by centrifugation and resuspended in PBS, followed by three cycles of liquid nitrogen freeze–thaw lysis. The lysates were centrifuged at 12,000 *g* for 15 min at 4 °C to collect the supernatant. For thermal shift experiments, supernatants were incubated with an equal volume of DMSO or CPD6 (final concentration 500 μM) at room temperature for 2 h; parallel experiments with MELK-8a as a positive control were performed using the identical protocol and compound concentration. Each lysate was then aliquoted into eight equal portions and heated individually at 45, 50, 55, 60, 65, 70, 75, and 80 °C for 5 min, followed by cooling at room temperature for 3 min. After centrifugation at 12,000× *g* for 20 min at 4 °C, the supernatants were collected and mixed with loading buffer for subsequent Western blot analysis.

### 2.17. Transfection

MDA-MB-468 and H1299 cells were transfected with the pCMV-MELK(human)-3×FLAG-SV40-Neo human MELK overexpression plasmid (MiaoLing Plasmid Sharing Platform, Wuhan, China, Cat. No. P36781) or corresponding empty vector control using Lipofectamine™ 3000 transfection reagent (Invitrogen, Carlsbad, CA, USA) according to the manufacturer’s instructions.

## 3. Results and Discussion

### 3.1. Overview of the ML-Based Virtual Screening Workflow

**Figure 1:**
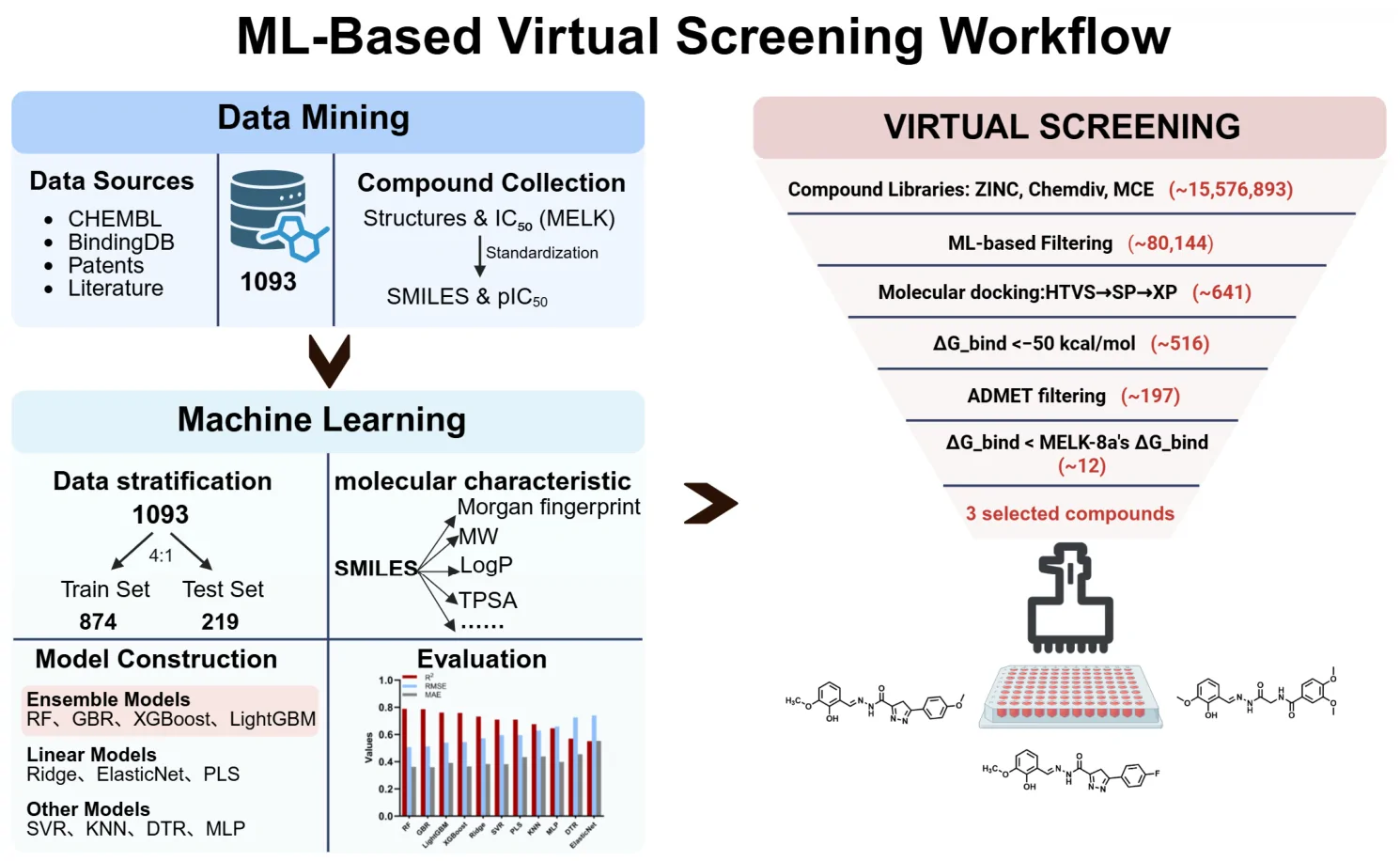
Schematic workflow of the machine learning-driven virtual screening pipeline for novel MELK inhibitors. Figure 1 illustrates the ML-driven virtual screening workflow for MELK inhibitors. A total of 1093 experimentally validated MELK inhibitors were collected from public databases, patents, and literature, split into training (874) and test (219) sets at a 4:1 ratio. Eleven regression models were constructed, with four top-performing tree-based ensemble models (RF, GBR, XGBoost, LightGBM) selected for large-scale screening. A 15,576,893-compound library was filtered through a multi-stage funnel: ML-based pIC_50_ prediction (~80,144 retained), sequential HTVS-SP-XP molecular docking (~641 retained), MM/GBSA binding free energy calculation (ΔG_bind < −50 kcal/mol, ~516 retained), drug-likeness filtering (~197 retained), and affinity-based prioritization, ultimately yielding 3 representative candidate compounds for further experimental validation. Created with BioRender.com.

Figure 1 outlines the overall workflow of the machine learning-driven virtual screening strategy identified in this study. The entire process encompasses data mining, model construction, multi-stage virtual screening, and final candidate selection.

### 3.2. Performance Evaluation of Machine Learning Models

**Figure 2:**
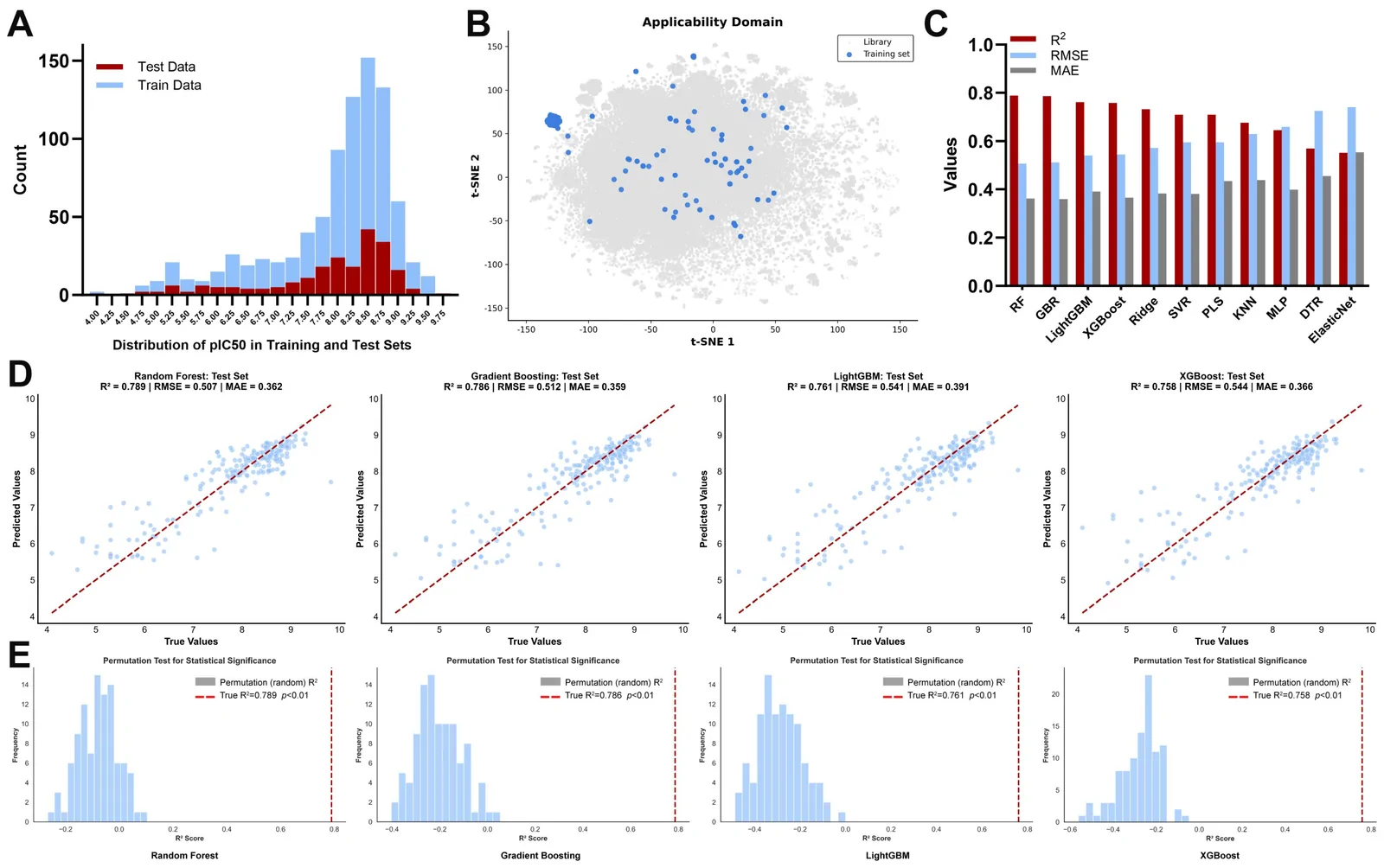
Performance evaluation of machine learning models for pIC_50_ prediction. Figure 2 summarizes the model validation results. (**A**) Comparison of pIC_50_ distributions between the training and test sets. (**B**) t-SNE visualization of the chemical space for the training set (blue dots) and virtual library (gray dots), demonstrating a reasonable applicability domain. (**C**) Comparative performance metrics (R^2^, RMSE, MAE) across 11 regression models. (**D**) Parity plots of the top four ensemble models (Random Forest, GBR, LightGBM, XGBoost) showing predicted vs. experimental pIC_50_ values on the test set. The diagonal dashed line represents the line of perfect prediction (y = x). (**E**) Permutation tests confirming the statistical significance of the four optimal models.

A total of 1093 unique MELK inhibitors were retrieved from the ChEMBL database, three patents, and twelve published studies. All IC_50_ values were converted to pIC_50_, resulting in a continuous activity range spanning from 4.0 to 9.75 that captures a broad spectrum of inhibitory potency. The entire dataset was randomly split into a training set and an independent test set at a ratio of 4:1. As shown in Figure 2A, the pIC_50_ distributions of the two subsets were highly consistent, thereby eliminating distributional bias and ensuring the reliability of subsequent model evaluations.

To initially verify the structural coverage of the training set within the virtual screening library, t-distributed stochastic neighbor embedding (t-SNE) was employed to visualize the chemical space distribution (Figure 2B). The results confirmed that the training set molecules were well embedded within the chemical space covered by the 15.58 million virtual library, indicating a reasonable applicability domain and laying a foundation for subsequent virtual screening.

The predictive performance of 11 distinct regression algorithms was systematically assessed on the test set (Figure 2C,D). Among all models, four tree-based ensemble models achieved markedly superior predictive accuracy: Random Forest (*R*^2^ = 0.789, RMSE = 0.507), GBR (*R*^2^ = 0.786, RMSE = 0.512), LightGBM (*R*^2^ = 0.761, RMSE = 0.541), and XGBoost (*R*^2^ = 0.758, RMSE = 0.558). Detailed quantitative metrics derived from 10-fold cross-validation are summarized in Table S2. Furthermore, permutation testing was conducted to validate the statistical significance of the optimal model (Figure 2E). All randomly permuted models exhibited drastically lower R^2^ values with no overlap relative to the original model, confirming that the predictive performance was statistically significant and not derived from random chance. Based on the above validation results, the four optimal ensemble models were selected for subsequent large-scale virtual screening.

### 3.3. Virtual Screening Results

Using the four top-performing models, we predicted the pIC_50_ values of 15,576,893 compounds from the ZINC (Drug-like and Lead-like) [27], Chemdiv (D001), and MCE (HY-L901P) databases. Compounds with predicted pIC_50_ values greater than 7 in at least three models were considered potential MELK inhibitors. After the initial screening, a total of 80,144 candidate compounds were obtained. These compounds were subsequently subjected to molecular docking and binding free energy calculations to further evaluate their binding affinities.

### 3.4. Molecular Docking and Binding Free Energy Analysis

A total of 80,144 candidate compounds were subjected to sequential docking using HTVS, SP, and XP protocols, with the top 20% of compounds retained at each stage based on docking scores, yielding 641 top-ranked compounds. Subsequent MM/GBSA rescoring was performed, and 516 compounds with computed ΔG_bind values more favorable than −50 kcal/mol were retained as an enriched subset for further analysis.

### 3.5. ADMET Property Prediction

Comprehensive ADMET profiles were systematically predicted for all 516 compounds using ADMETlab, covering a full set of absorption, distribution, metabolism, excretion, and toxicity-related endpoints, along with additional physicochemical and safety-associated descriptors. For candidate prioritization, filtering was conducted exclusively using four widely accepted drug-likeness rules: Lipinski’s Rule of Five, Pfizer 3/75 rule, GSK 4/400 rule, and Golden Triangle criterion. A total of 197 compounds complied with all four drug-likeness criteria. Key representative ADMET descriptors for the final 12 compounds are summarized in Table S3.

### 3.6. Binding Free Energy Screening and Structural Novelty Analysis

To further narrow the candidate set, the well-characterized MELK inhibitor MELK-8a was included as a reference compound. Its re-docked pose was rescored with Prime MM/GBSA, yielding a computed ΔG_bind of −77.11 kcal/mol, which was used as a reference threshold for relative ranking. We then compared the MM/GBSA scores of the 197 ADMET-filtered compounds against this reference and prioritized 12 compounds whose computed ΔG_bind values were more favorable than that of the re-docked MELK-8a pose (Tables S4 and S5). We emphasize that these MM/GBSA differences are used only as a relative prioritization metric within the computational workflow and are not interpreted as evidence that these compounds bind MELK more tightly than MELK-8a in a biochemical or biophysical assay, given the approximate nature and multi-kcal/mol uncertainty of MM/GBSA scoring.

To assess the structural novelty of these candidate compounds, we calculated ECFP4 fingerprints and compared each molecule to the most structurally similar known MELK inhibitors (Table S6). Tanimoto similarity indices for the 12 prioritized compounds ranged from 0.231 to 0.305 relative to previously reported MELK inhibitors, indicating that they occupy regions of chemical space distinct from established MELK inhibitor chemotypes [1,15,16,19,20,21,22,23,24,25,26]. This high degree of structural novelty is notable because many existing MELK inhibitors derive from a relatively small number of scaffolds; discovery of chemically distinct starting points provides fresh vectors for structure–activity relationship optimization and may yield compounds with differentiated selectivity or physicochemical profiles. These 12 compounds therefore represent chemotypes that, according to the computational rescoring, adopt energetically plausible binding poses in the MELK kinase domain and warrant experimental evaluation. Structural novelty alone does not imply improved target affinity, which must be established experimentally.

To facilitate experimental validation, compound selection was refined based on commercial availability. Among the twelve candidate MELK-binding compounds, five compounds from the ZINC database were not purchasable, whereas seven Chemdiv-derived compounds were accessible and categorized into two major scaffolds. From these, three representative compounds (CPD4, CPD6, and CPD10) were selected for subsequent experimental evaluation.

### 3.7. Docking Analysis of CPD4, CPD6, and CPD10 with MELK

**Figure 3:**
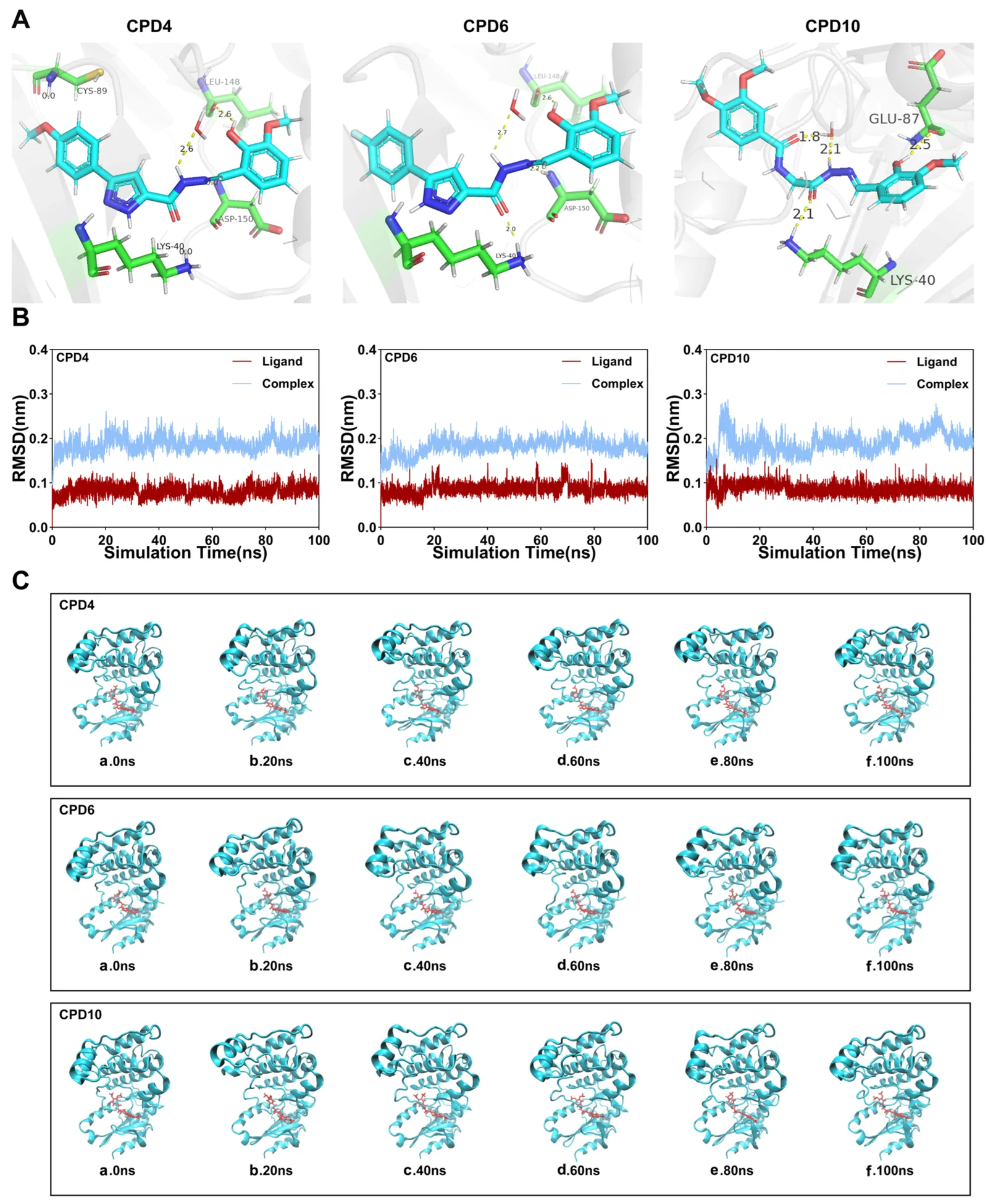
Binding interactions and molecular dynamics stability of the three selected candidate compounds. (**A**) 3D binding conformations of CPD4, CPD6, and CPD10 in the MELK active site. Hydrogen bonds (yellow dashed lines) between ligands and key residues (Lys40, Asp150, Cys89, Glu87, etc.) are labeled with distances (nm). (**B**) RMSD profiles of the ligand (red) and protein–ligand complex (blue) over 100 ns MD simulations, confirming the structural stability of each system. (**C**) Structural superimposition of the protein–ligand complexes at different time points (0, 20, 40, 60, 80, and 100 ns) during the simulation, demonstrating the overall conformational stability of the protein (cyan) and the consistent binding pose of the three compounds (red).

The docking conformations of CPD4, CPD6, and CPD10 with MELK were visualized to investigate their binding interactions (Figure 3A). All three compounds occupied the ATP-binding pocket, forming multiple stabilizing contacts. Specifically, hydrogen bonds were observed between the ligands and key residues, such as Lys40 and Glu87, while additional hydrophobic interactions further stabilized the complexes. The overall binding modes suggest that these compounds can be accommodated within the ATP pocket and form plausible interactions with key residues; the stability of these poses was further assessed by molecular dynamics simulations.

### 3.8. Molecular Dynamics Simulation Analysis

To further verify the binding stability of CPD4, CPD6, and CPD10 with the MELK protein, 100 ns molecular dynamics (MD) simulations were conducted. The root mean square deviation (RMSD) profiles, as shown in Figure 3B, were utilized to assess the structural stability of each complex system over time. The RMSD analysis revealed that both the protein backbone and the protein–ligand complex of CPD4, CPD6 and CPD10 maintained low and stable values throughout the simulation, remaining below approximately 0.3 nm, which indicated good overall structural integrity of the complexes. Structural superimposition of protein–ligand complexes sampled at 20 ns intervals across the 100 ns trajectory further confirmed that the bound ligand pose remained consistent over time, with no large conformational drift of the protein backbone or ligand binding orientation (Figure 3C).

Consistent with previously reported molecular dynamics studies of kinase-inhibitor complexes, protein backbone and protein–ligand complex RMSD values remaining below 0.3 nm over 100 ns simulations are generally interpreted as indicating that the modeled binding pose does not undergo large conformational drift over the simulation timescale [22,24]. For CPD6, the docking-predicted pose is positioned within the canonical ATP-binding pocket, with predicted polar contacts to Lys40, Asp150 and Leu148. This modeled pose provides a reasonable working hypothesis for future structural characterization and structure-based optimization. We emphasize that stable RMSD trajectories and favorable MM/GBSA scores do not constitute experimental proof of binding affinity or binding mode; co-crystallization or direct biophysical binding assays will be required to experimentally validate this predicted binding mode in future work.

### 3.9. In Vitro Antiproliferative Activity of Candidate Compounds

**Figure 4:**
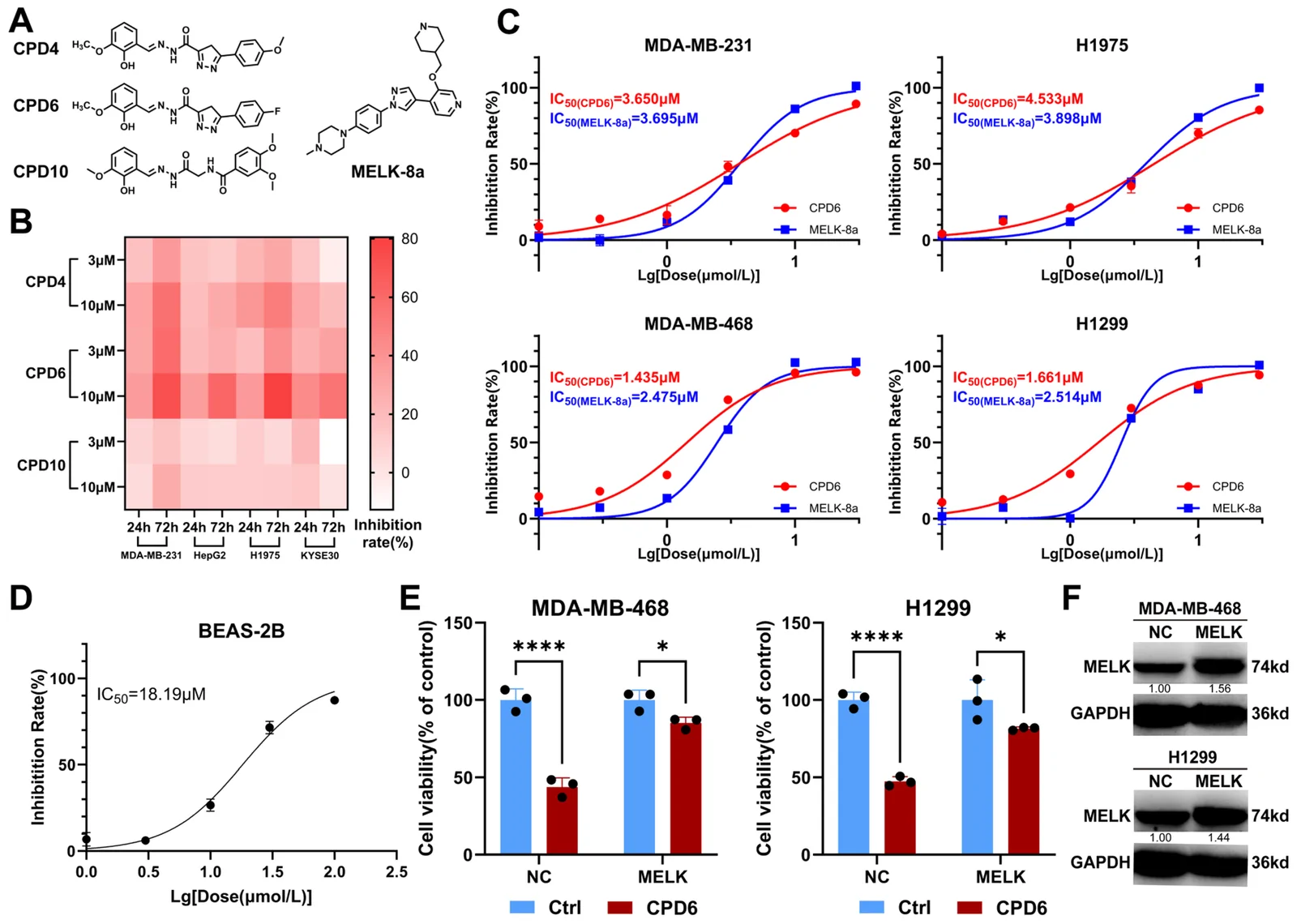
Chemical structures and in vitro antiproliferative activities of selected candidate compounds. Figure 4 presents the structural and cellular activity profiles of the candidate compounds. (**A**) Chemical structures of CPD4, CPD6, CPD10, and the positive control MELK-8a. (**B**) Heatmap showing growth inhibition rates of four cancer cell lines (MDA-MB-231, HepG2, H1975, KYSE30) after treatment with the three compounds at 3 μM and 10 μM for 24 or 72 h. (**C**) Dose–response curves and corresponding IC_50_ values of CPD6 and MELK-8a in MDA-MB-231, H1975, MDA-MB-468, and H1299 cell lines. (**D**) Dose–response curve and IC_50_ value of CPD6 in BEAS-2B normal bronchial epithelial cells after 72 h treatment. (**E**) Cell viability of empty vector (NC) or MELK-overexpressing MDA-MB-468 and H1299 cells treated with DMSO or 1.5 μM CPD6 for 72 h. (**F**) Western blot validation of MELK overexpression in the indicated cell lines. n = 3 independent biological replicates. Statistical significance: * *p* < 0.05, **** *p* < 0.0001.

The in vitro antiproliferative activities of CPD4, CPD6, and CPD10 were first evaluated by single-concentration screening in four tumor cell lines spanning four indications: MDA-MB-231 (TNBC), H1975 (NSCLC), HepG2 (hepatocellular carcinoma), and KYSE30 (esophageal squamous-cell carcinoma). Figure 4A shows the chemical structures of the three candidate compounds and the positive control MELK-8a. Screening was performed at 3 μM and 10 μM for 24 h and 72 h, and growth inhibition rates are presented as a heatmap in Figure 4B. CPD6 exhibited the most potent activity among the three compounds. At 10 μM and 72 h, CPD6 inhibited proliferation by more than 70% in MDA-MB-231 and H1975, whereas activity in HepG2 and KYSE30 was only modest under the same conditions. CPD4 displayed moderate activity, and CPD10 showed minimal inhibition across the four lines. On the basis of this initial profiling, TNBC and NSCLC were prioritized for follow-up, while HepG2 and KYSE30 were not pursued further in the present study.

To further characterize CPD6 activity in the two responsive indications, two additional cell lines reported to express readily detectable MELK and widely used in prior MELK studies were introduced into the panel: MDA-MB-468 (TNBC) and H1299 (NSCLC) [3]. Full dose–response IC_50_ values for CPD6 and MELK-8a were then determined across the four TNBC and NSCLC lines. CPD6 showed IC_50_ values of 1.435 μM in MDA-MB-468 and 1.661 μM in H1299, which were more potent than those in MDA-MB-231 (3.650 μM) and H1975 (4.533 μM) (Figure 4C). MDA-MB-468 and H1299 were selected for subsequent colony formation, EdU incorporation, cell cycle, Western blot, and CETSA experiments. This choice was based on their higher sensitivity to CPD6, with IC_50_ values approximately two- to three-fold lower than those observed in MDA-MB-231 and H1975, as well as on literature documenting readily detectable MELK expression and their prior use as model systems in MELK functional studies [3,38]. Focusing on one representative line per indication also allowed a balanced comparison between TNBC and NSCLC phenotypes in the downstream mechanistic assays. We note that a formal quantitative correlation between baseline MELK protein abundance and CPD6 sensitivity across the full six-cell panel was not performed in the present study and will be addressed in future work.

To validate these findings, the well-characterized MELK inhibitor MELK-8a was used as a positive control. Its IC_50_ values were 2.475 μM in MDA-MB-468 and 2.514 μM in H1299, both higher than those of CPD6 (Figure 4C). These results demonstrate that CPD6 exerts low-micromolar antiproliferative activity in both TNBC and NSCLC lines, with IC_50_ values comparable to or modestly lower than those of the reference compound MELK-8a under the same assay conditions. CPD6 showed an IC_50_ of 18.19 μM in the non-cancerous human bronchial epithelial cell line BEAS-2B after 72 h treatment, approximately 10-fold higher than its IC_50_ in H1299 lung adenocarcinoma cells, indicating a preliminary selectivity window and absence of broad non-specific cytotoxicity (D). In both MDA-MB-468 and H1299 cells, transient overexpression of MELK significantly attenuated CPD6-induced proliferation inhibition at 1.5 μM concentrations, further supporting that the antiproliferative activity of CPD6 is partially MELK-dependent (Figure 4E,F).

### 3.10. CPD6 Engages MELK in Cells and Reduces MELK Protein Levels

**Figure 5:**
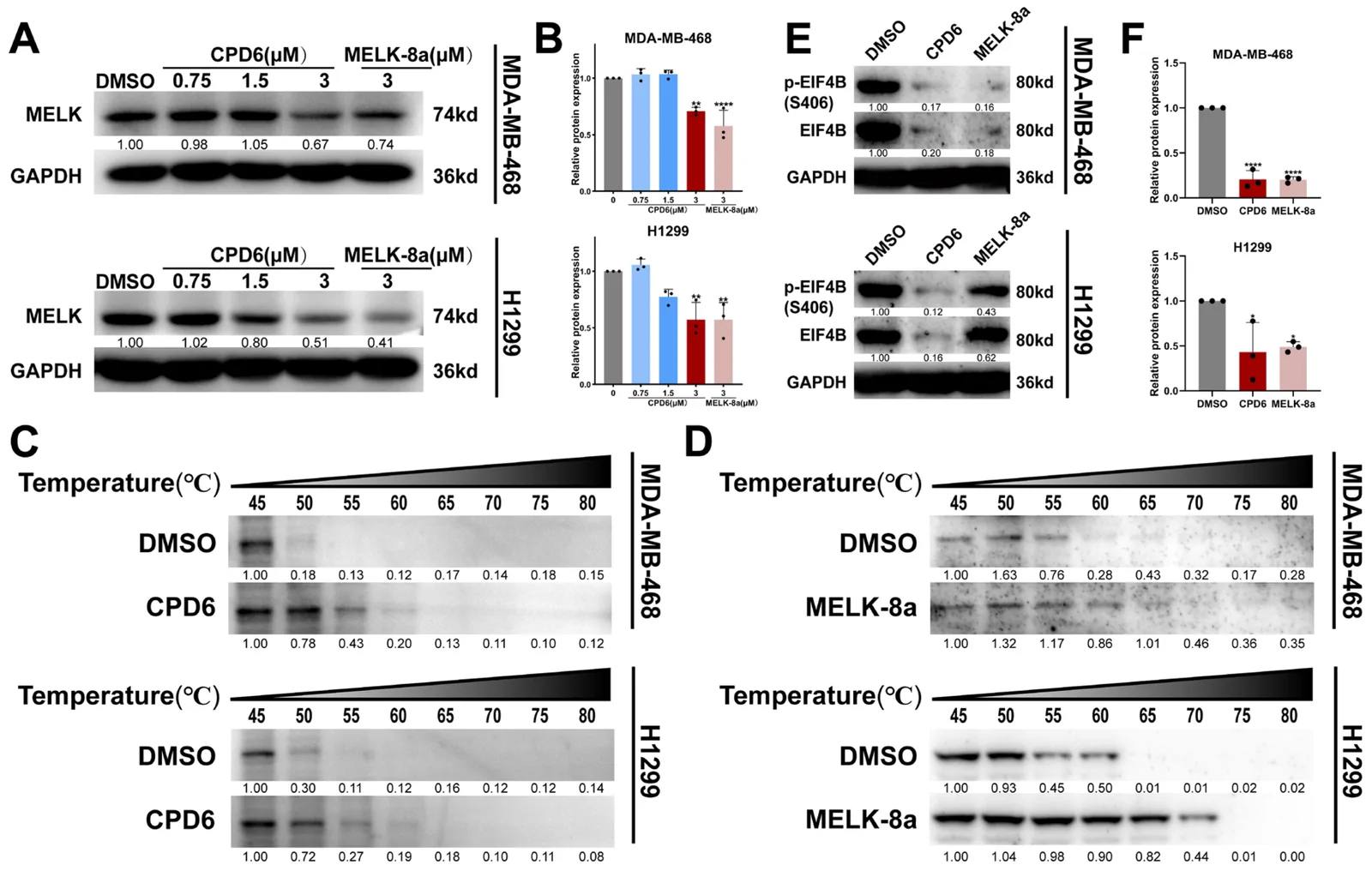
Effects of CPD6 on MELK protein expression, substrate phosphorylation, and thermal stability in MDA-MB-468 and H1299 cells. (**A**) Western blot analysis of MELK protein expression in MDA-MB-468 and H1299 cells treated with DMSO, CPD6 (0.75, 1.5, or 3 μM), or 3μM MELK-8a. (**B**) Quantification of relative MELK protein levels from panel A. (**C**) CETSA thermal stability profiles of MELK in cells treated with DMSO or CPD6 across the indicated temperature gradient. (**D**) CETSA thermal stability profiles of MELK in cells treated with DMSO or MELK-8a positive control. (**E**) Western blot of phosphorylated EIF4B (Ser406) and total EIF4B in treated cells. (**F**) Quantification of relative p-EIF4B (Ser406) levels from panel C. Statistical significance: * *p* < 0.05, ** *p* < 0.01, **** *p* < 0.0001.

Expression of the MELK protein in cells treated with increasing concentrations of CPD6 was checked by Western blot; full uncropped original blots for all Western blot results in this study are provided in Supplementary File S1. The results showed a dose-dependent decrease in MELK expression (Figure 5A,B). To further investigate the direct interaction between CPD6 and MELK, the Cellular Thermal Shift Assay was carried out, which relies on the principle that small-molecule binding enhances the thermal stability of target proteins. CPD6-treated lysates showed markedly increased MELK thermal stability compared with DMSO-treated controls in both MDA-MB-468 and H1299 backgrounds (Figure 5C), with MELK remaining in the soluble fraction at temperatures where the DMSO-treated protein had fully precipitated. Because this assay is performed in cell lysates in the absence of intact cellular processes such as transcription, translation, cell cycle progression, or cell death, the observed thermal shift provides direct biochemical evidence that CPD6 physically engages the MELK protein and stabilizes it against thermal denaturation, rather than reflecting an indirect consequence of growth arrest or cellular stress. CETSA experiments with the selective MELK inhibitor MELK-8a as a positive control produced a thermal stabilization effect on the MELK protein, confirming the assay system was functioning as intended (Figure 5D). To assess effects on MELK catalytic activity, we examined phosphorylation of EIF4B at Ser406, a well-characterized direct MELK substrate. Western blot analysis showed a clear reduction in p-EIF4B (Ser406) levels following CPD6 treatment, and MELK-8a produced a similar reduction in p-EIF4B levels (Figure 5E,F).

These findings are relevant to the ongoing debate regarding MELK tractability as an anticancer target. Although landmark CRISPR knockout studies have suggested that MELK is not essential for basal proliferation of triple-negative breast cancer cells [18], such conclusions do not negate the potential antitumor utility of small molecules that engage MELK and modulate its function or protein abundance [13]. We note that CETSA performed in cell lysates confirms physical binding but does not on its own demonstrate on-target functional inhibition, nor does it rule out engagement of additional kinase or non-kinase targets. Comprehensive kinome-wide selectivity profiling, direct enzymatic inhibition assays, and biophysical binding measurements will be required to fully characterize CPD6′s target spectrum and clarify its mode of action, which may include ATP-competitive inhibition, allosteric modulation, or indirect effects on MELK protein stability.

### 3.11. CPD6 Inhibits Tumor Cell Proliferation

**Figure 6:**
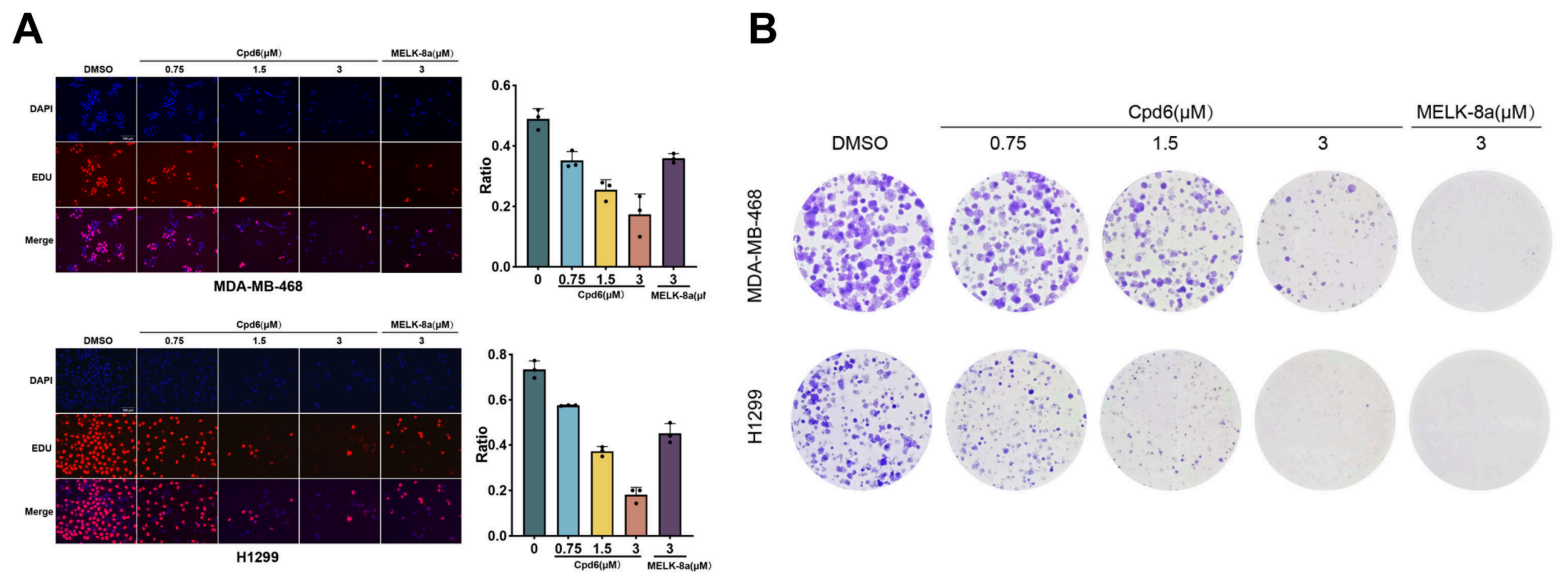
Effects of CPD6 on proliferation of MDA-MB-468 and H1299 cells. (**A**) EdU incorporation assay. MDA-MB-468 and H1299 cells were treated with DMSO, CPD6 (0.75, 1.5, or 3 μM), or 3 μM MELK-8a. EdU-positive cells (red) and DAPI-stained nuclei (blue) were visualized by fluorescence microscopy. The ratio of EdU-positive cells was quantified. (**B**) Colony formation assay. Cells were treated with DMSO, CPD6 (0.75, 1.5, or 3 μM), or 3 μM MELK-8a, and colony formation was assessed by crystal violet staining.

To further validate the in vitro antiproliferative activity of CPD6 in sensitive cell lines, colony formation assays and EdU incorporation assays were carried out. CPD6 inhibited DNA synthesis and colony formation of MDA-MB-468 and H1299 cells in a dose-dependent manner (Figure 6A,B).

### 3.12. CPD6 Induces G2/M Cell Cycle Arrest

**Figure 7:**
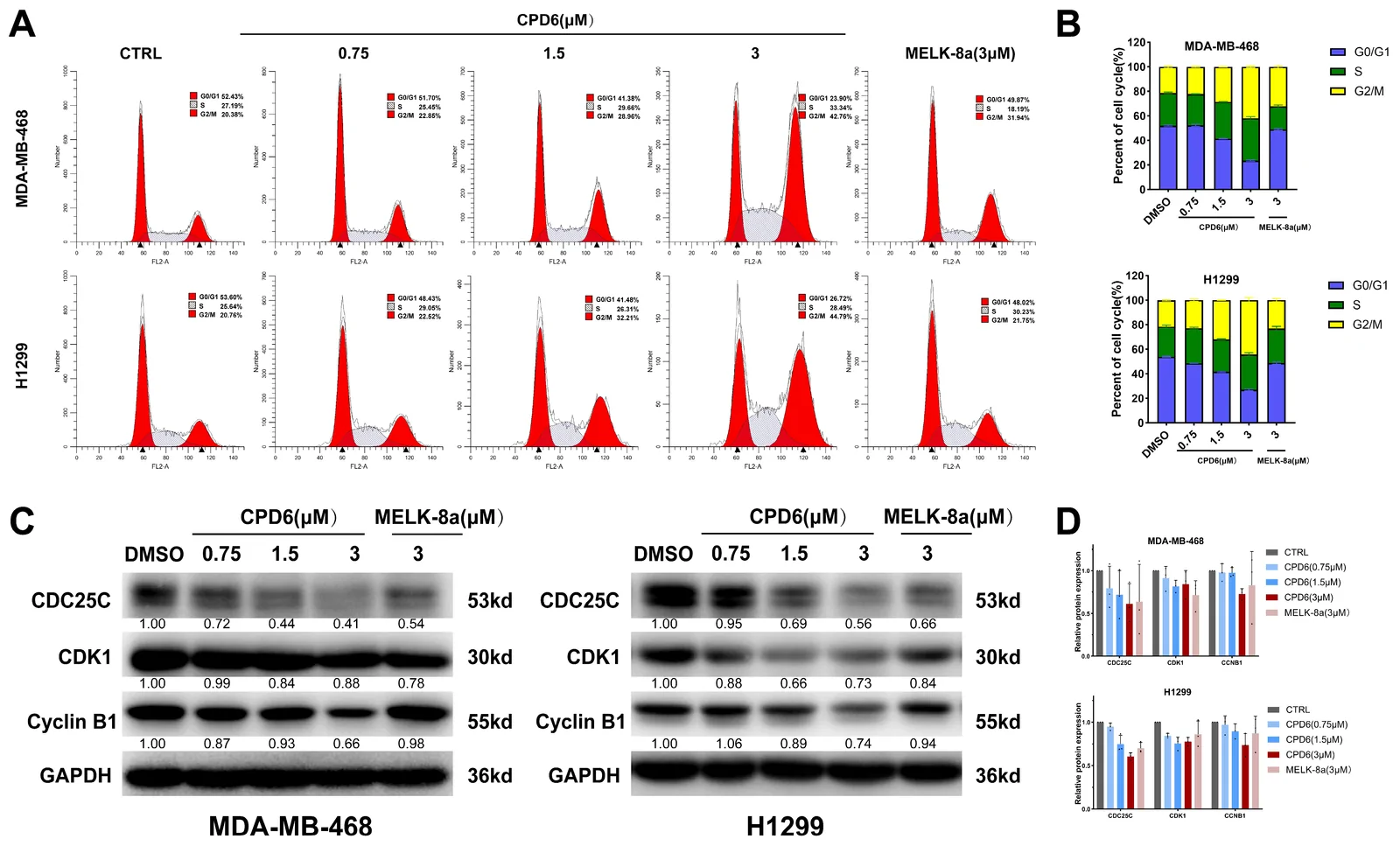
CPD6 induces cell cycle arrest and regulates cell cycle-related proteins in MDA-MB-468 and H1299 cells. (**A**) Flow cytometric analysis of cell cycle distribution in MDA-MB-468 and H1299 cells treated with DMSO (control), CPD6 (0.75, 1.5, or 3 μM), or MELK-8a (3 μM). (**B**) Quantification of the percentage of cells in G0/G1, S, and G2/M phases from panel A. (**C**) Western blot analysis of cell cycle-related proteins CDC25C, CDK1, and Cyclin B1 in MDA-MB-468 and H1299 cells treated with DMSO, CPD6 (0.75, 1.5, or 3 μM), or MELK-8a (3 μM). (**D**) Quantification of relative protein levels from panel (**C**).

The results from flow cytometry showed that CPD6 induced G2/M cell cycle arrest (Figure 7A,B), and Western blotting revealed a dose-dependent decrease in total CDC25C, CDK1 (CDC2), and Cyclin B1 levels (Figure 7C,D). Taken together, these data indicate that CPD6 blocks the G2-to-M transition by downregulating the CDC25C–CDK1–CCNB1 axis, which likely contributes to its antiproliferative effect. This G2/M arrest phenotype is consistent with prior reports linking MELK function to mitotic progression, which demonstrate that MELK expression peaks during the G2/M phase of the cell cycle and that genetic MELK depletion or treatment with selective MELK inhibitors has been shown to delay mitotic entry via dysregulation of the CDK1/Cyclin B signaling axis [3,38]. The concomitant downregulation of CDC25C, CDK1, and Cyclin B1 at the protein level supports a model in which CPD6 blocks the G2-to-M transition at the level of the CDC25C-CDK1-Cyclin B1 core mitotic regulatory axis.

## 4. Conclusions

In this study, we established an integrated computational workflow combining machine learning-based activity prediction, hierarchical molecular docking, MM/GBSA rescoring, ADMET profiling, and 100 ns molecular dynamics simulation to screen approximately 15.58 million commercially available compounds for novel MELK-targeted inhibitors.

This campaign identified CPD6 as a structurally novel lead with low-micromolar antiproliferative activity against triple-negative breast cancer MDA-MB-468 cells and lung adenocarcinoma H1299 cells, preferential activity relative to non-cancerous bronchial epithelial cells, and potency exceeding that of the reference inhibitor MELK-8a under identical assay conditions.

Cellular thermal shift assay in cell lysates confirmed physical engagement between CPD6 and the MELK protein, and functional assays demonstrated dose-dependent inhibition of colony formation and DNA synthesis, along with G2/M cell cycle arrest mediated by downregulation of the CDC25C–CDK1–Cyclin B1 axis. CPD6 represents a promising cell-active lead for future MELK-focused hit-to-lead optimization, and the integrated screening pipeline established here provides an efficient, generalizable strategy for early-stage anticancer lead discovery.

## Data Availability

Relevant source code will be made publicly available on GitHub (https://github.com/). Additional raw data are available from the corresponding author on reasonable request.

## Supplementary Materials

The following supporting information can be downloaded at: https://www.mdpi.com/article/10.3390/cancers18203336/s1, File S1: Original Western Blots; Table S1: Information of antibodies; Table S2: Performance Metrics of Various Machine Learning Models Evaluated by 10-Fold Cross-Validation; Table S3: ADMET Prediction Results of 12 potential MELK compounds; Table S4: Basic information of candidate compounds; Table S5: Predicted pIC50 values, docking scores, and MMGBSA binding energy calculations for 13 compounds; Table S6: Chemical similarity between MELK hits and their closest ligands.
