# Supplementary material for: Machine Learning-Guided Virtual Screening Identifies a Novel MELK-Targeted Antiproliferative Lead Compound with G2/M Arrest Activity in Triple-Negative Breast Cancer and Lung Adenocarcinoma Cells
Source: Cancers (Basel). 2026 Oct 15;18(20):3336. doi: 10.3390/cancers18203336 (PMC13702308; doi:10.3390/cancers18203336)

Fig 4F

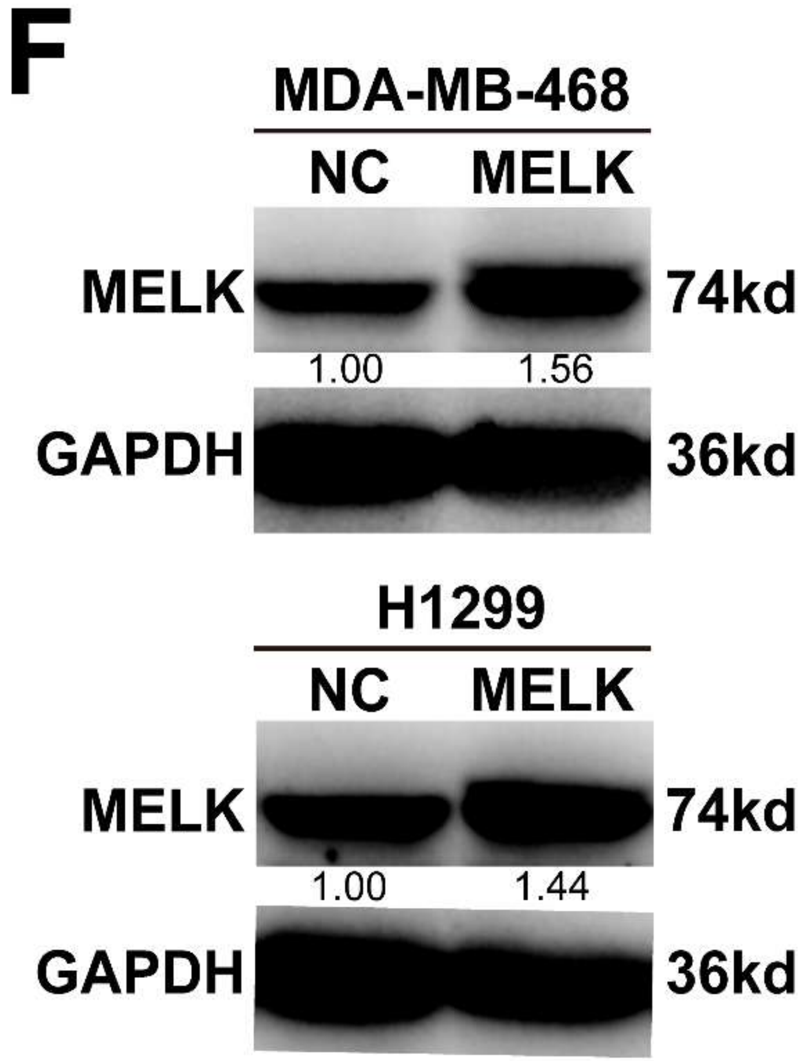
F
MDA-MB-468
NC
MELK
MELK
74kd
1.00
1.56
GAPDH
36kd
H1299
NC
MELK
MELK
74kd
1.00
1.44
GAPDH
36kd

Cell type:MDA-MB-468
Target protein: MELK
Sample ID:20
Corresponds to main text Figure 4F.
Red-framed bands are shown in the main text figure.

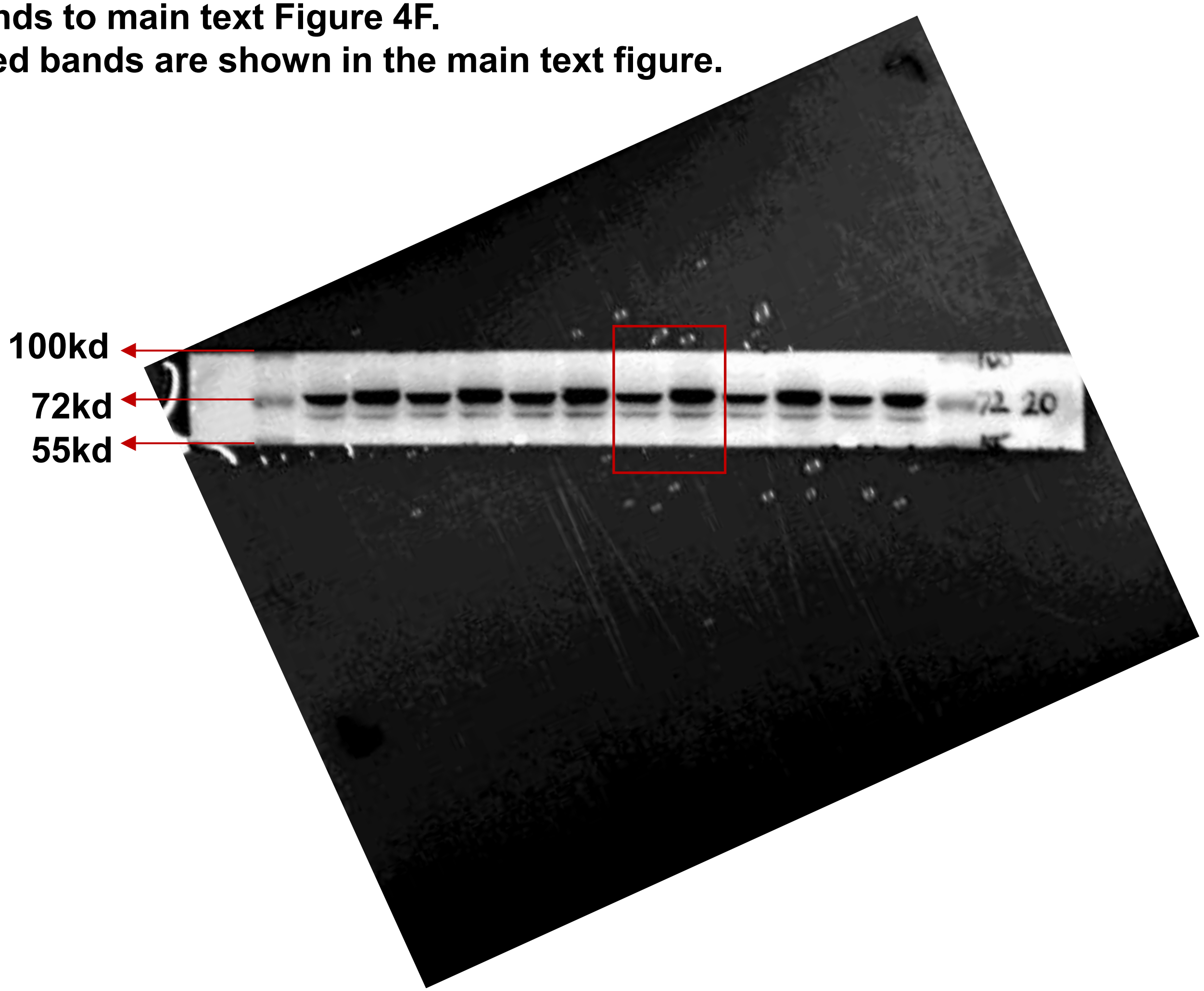

Cell type:MDA-MB-468
Target protein: gapdh
Sample ID:20
Corresponds to main text Figure 4F.
Red-framed bands are shown in the main text figure.

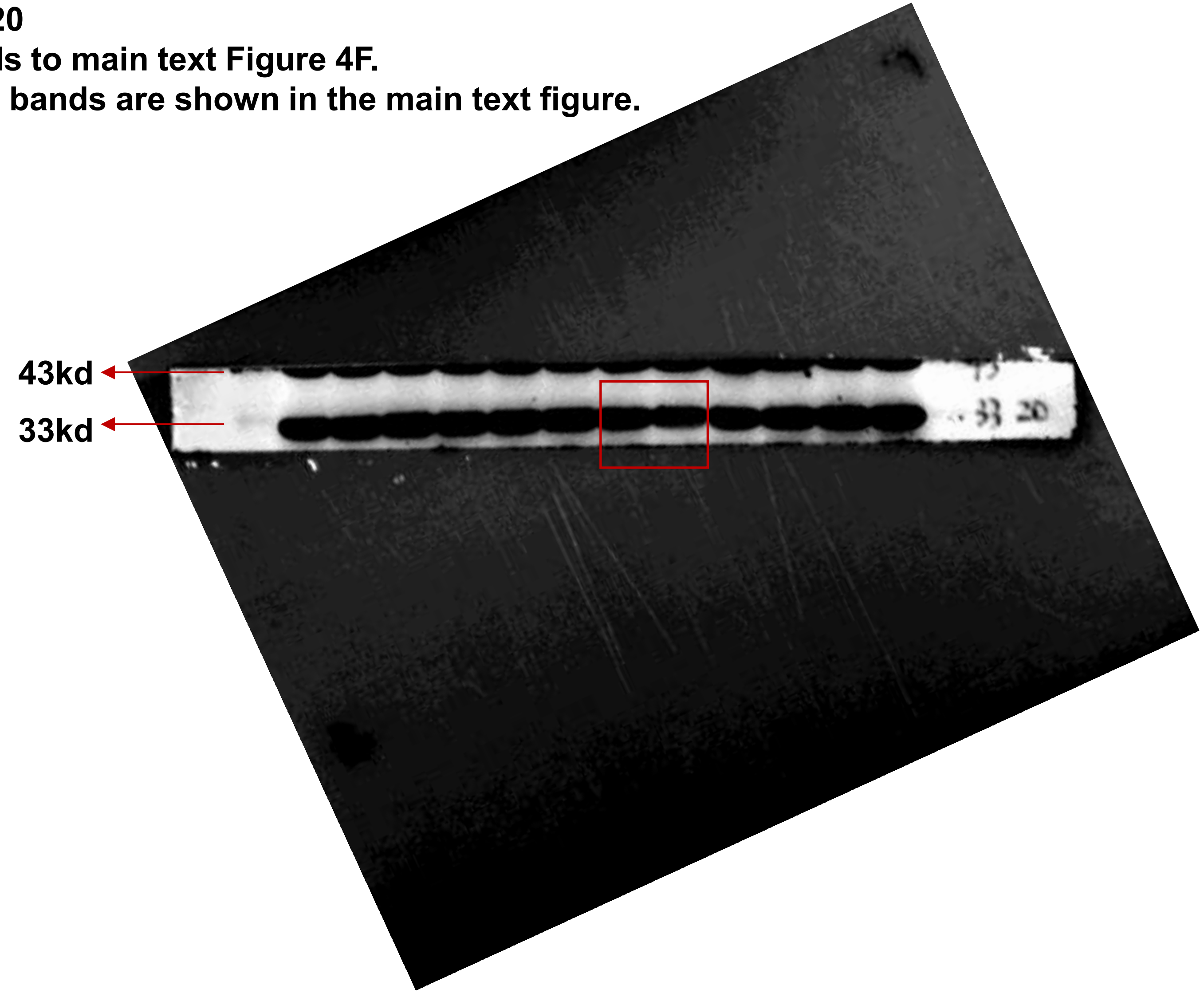

Cell type:H1299
Target protein: MELK
Sample ID:21
Corresponds to main text Figure 4F.
Red-framed bands are shown in the main text figure.

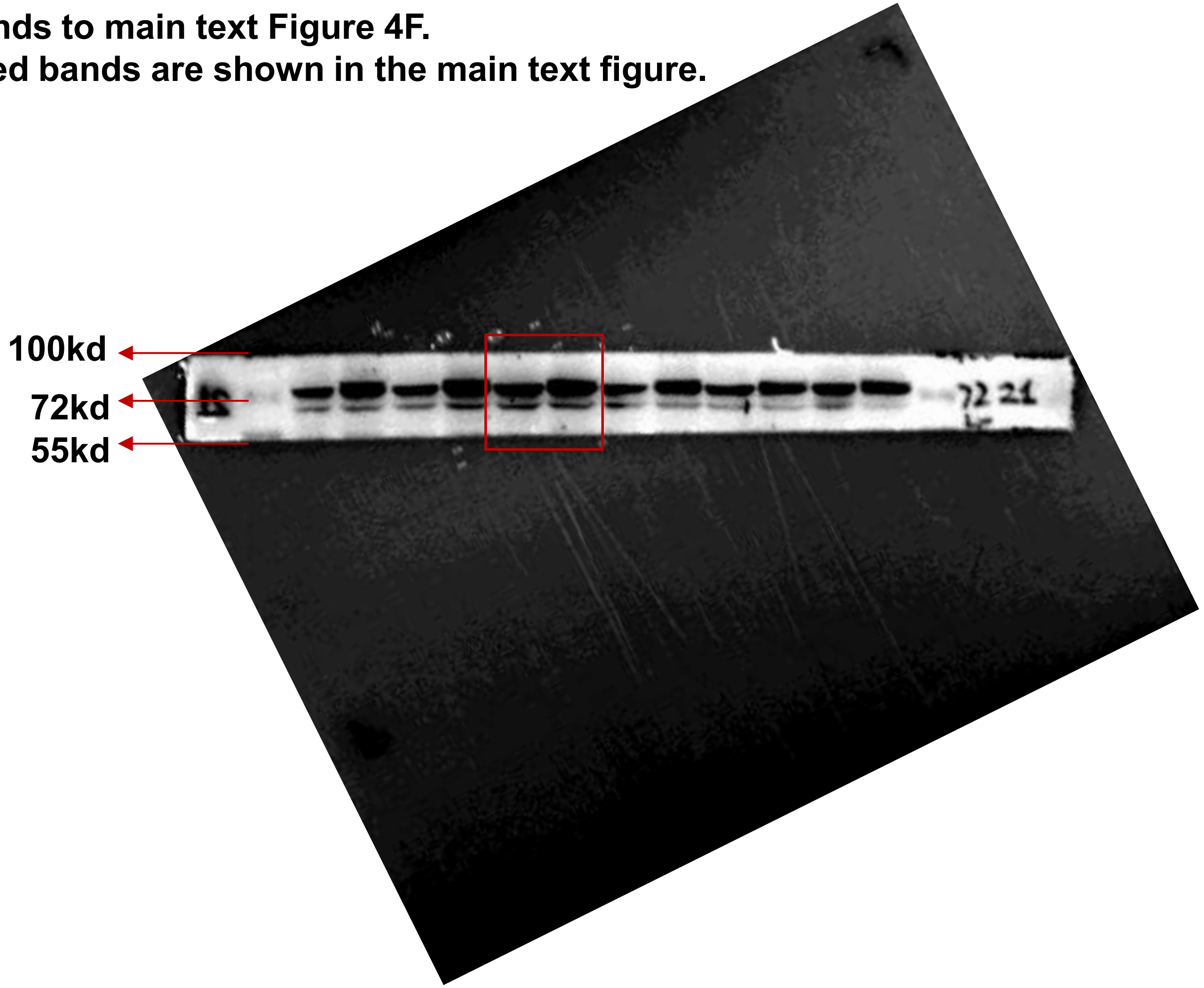

Cell type:H1299
Target protein: gapdh
Sample ID:21
Corresponds to main text Figure 4F.
Red-framed bands are shown in the main text figure.

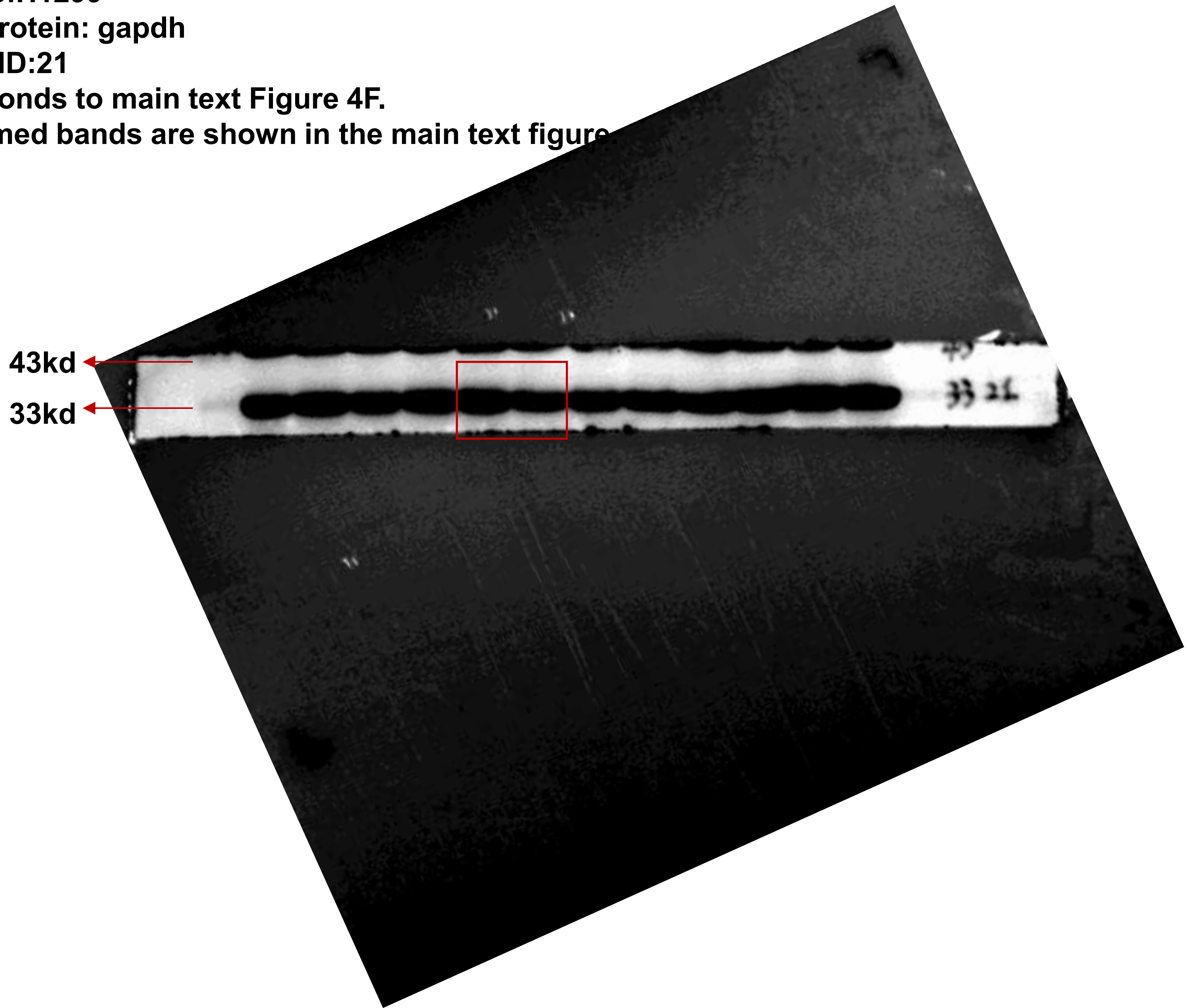

Fig 5A

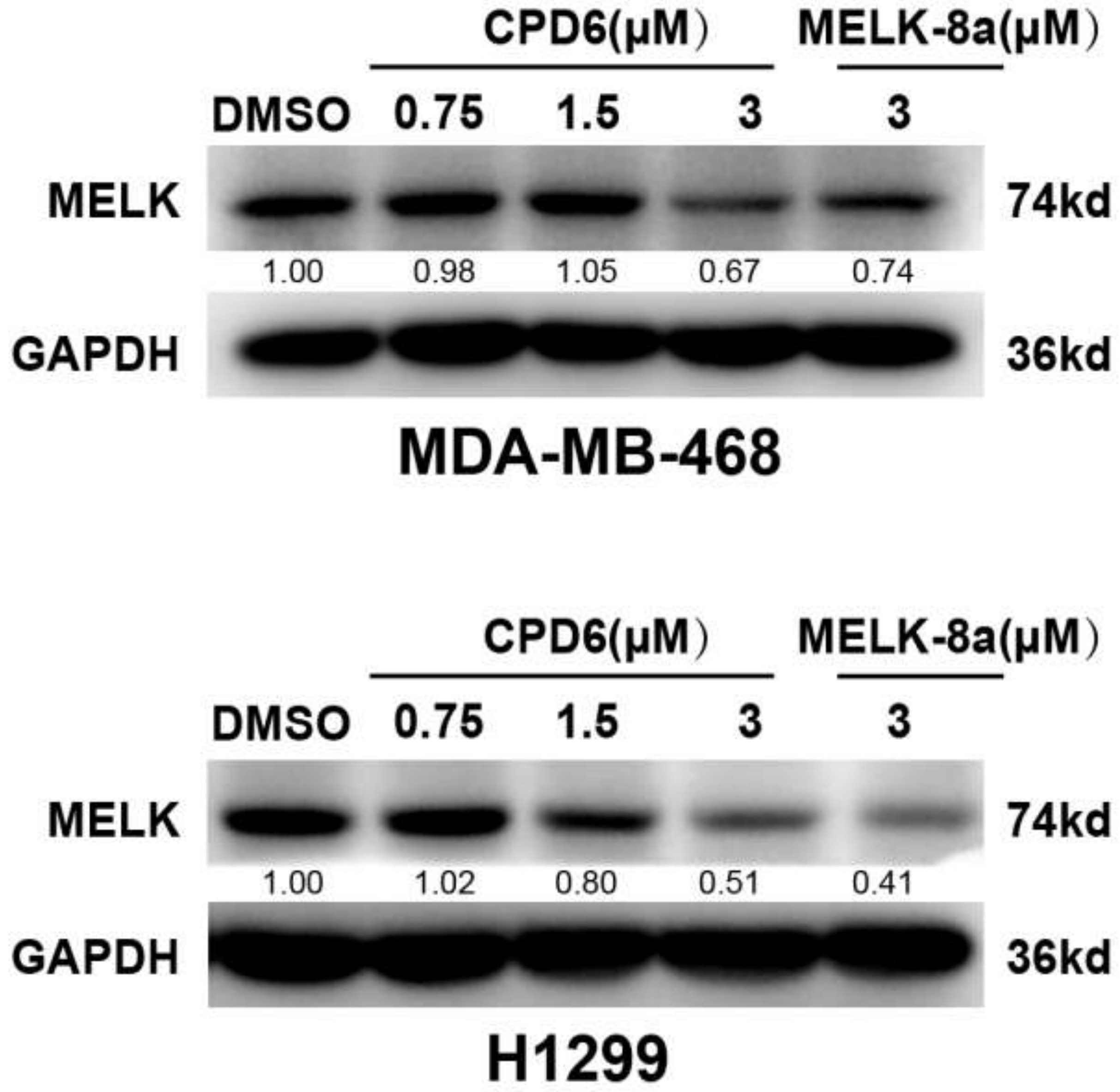

CPD6(μM)
MELK-8a(μM)
DMSO
0.75
1.5
3
3
MELK
74kd
1.00
0.98
1.05
0.67
0.74
GAPDH
36kd
MDA-MB-468
CPD6(μM)
MELK-8a(μM)
DMSO
0.75
1.5
3
3
MELK
74kd
1.00
1.02
0.80
0.51
0.41
GAPDH
36kd
H1299

Cell type:MDA-MB-468
Target protein:MELK
Sample ID:45
Corresponds to main text Figure 5A.
Red-framed bands are shown in the main text figure.

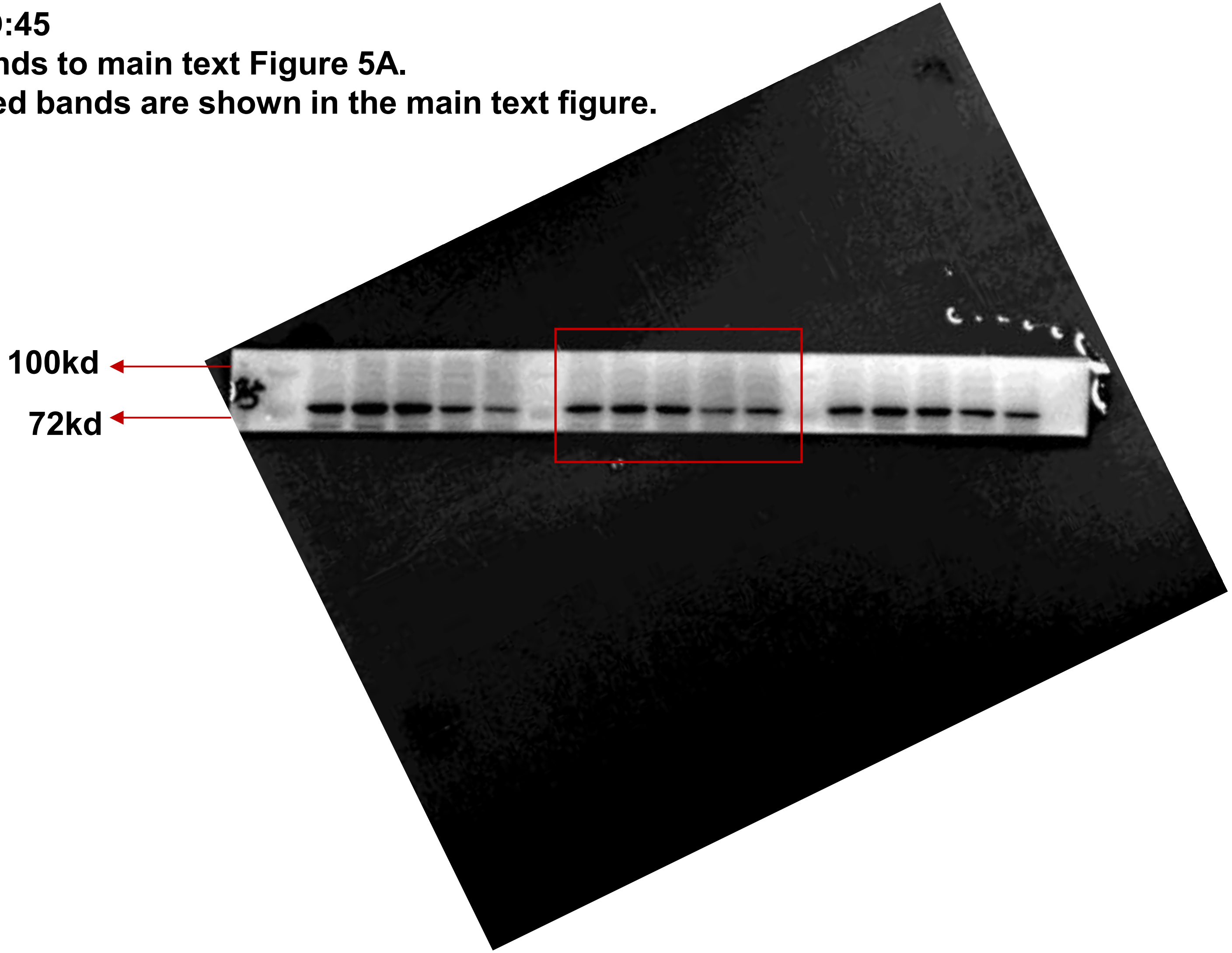

Cell type:MDA-MB-468
Target protein:GAPDH
Sample ID:45
Corresponds to main text Figure 5A.
Red-framed bands are shown in the main text figure.

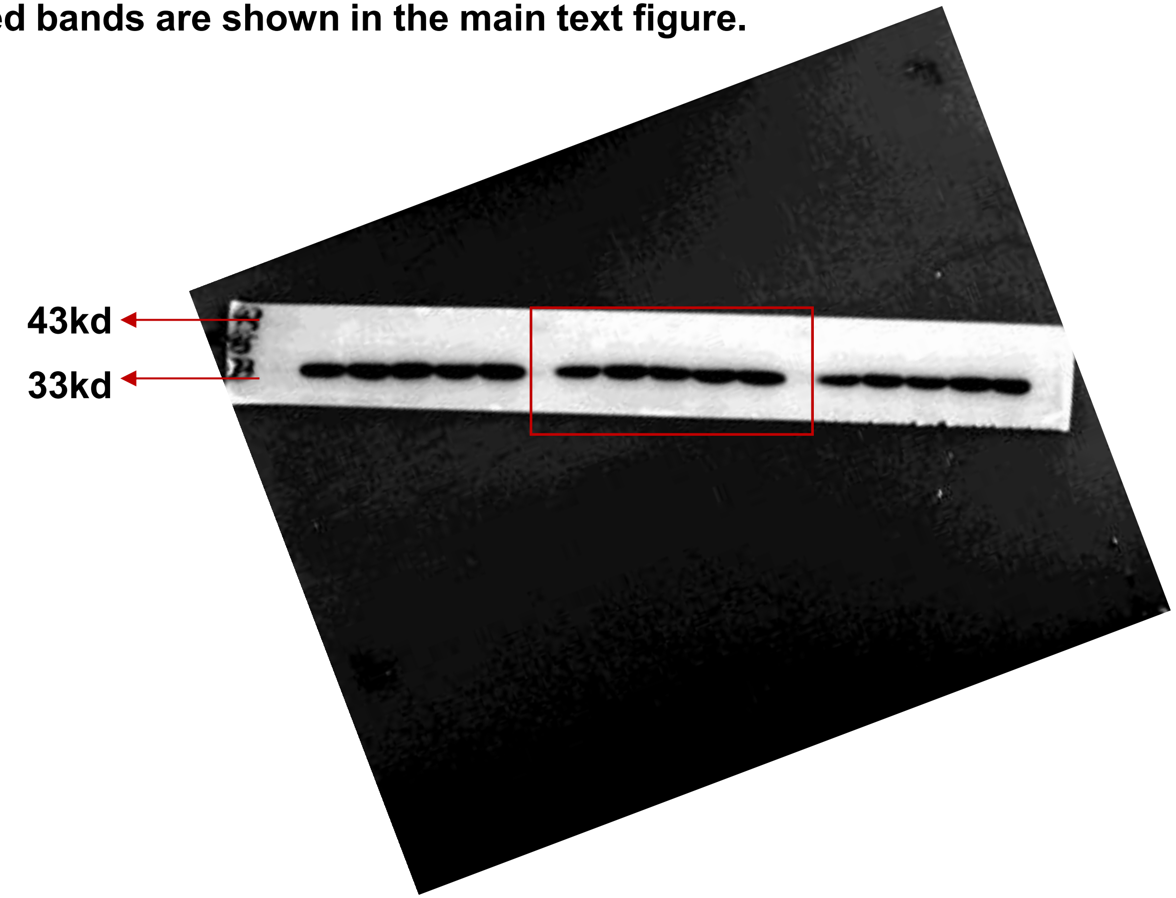

Cell type:H1299
Target protein:MELK
Sample ID:66
Corresponds to main text Figure 5A.
Red-framed bands are shown in the main text figure.

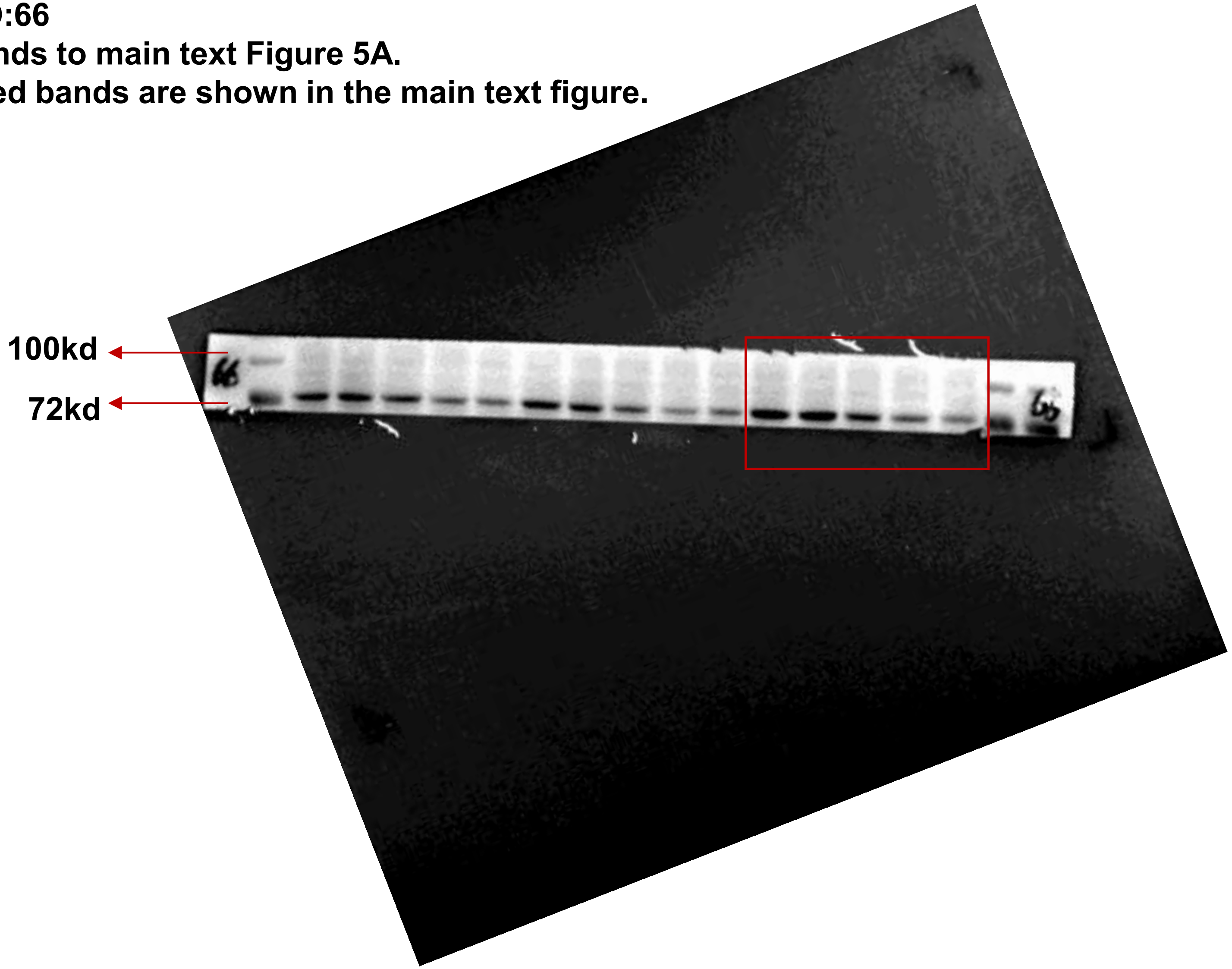

Cell type:H1299
Target protein:GAPDH
Sample ID:66
Corresponds to main text Figure 5A.
Red-framed bands are shown in the main text figure.

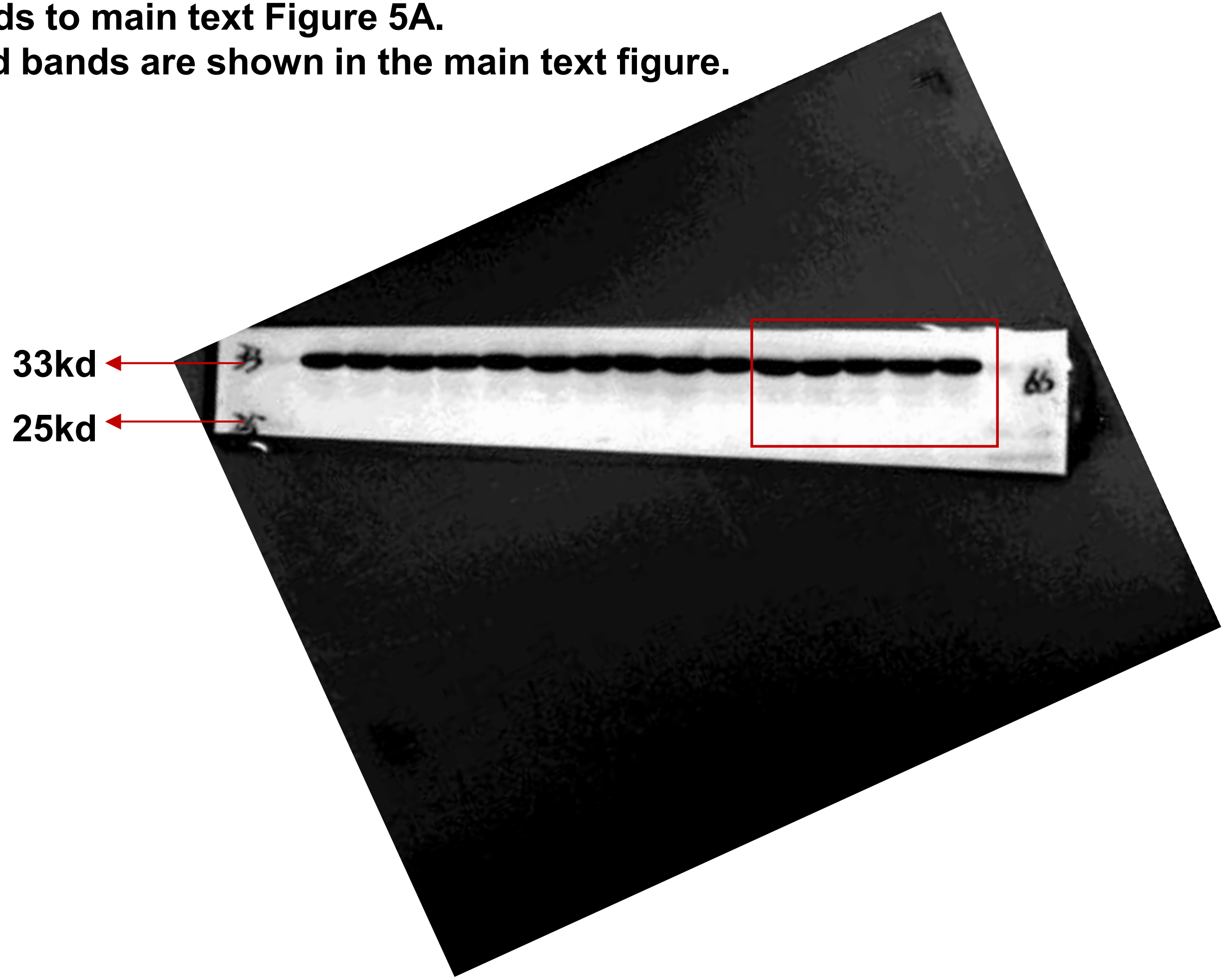

Fig 5C

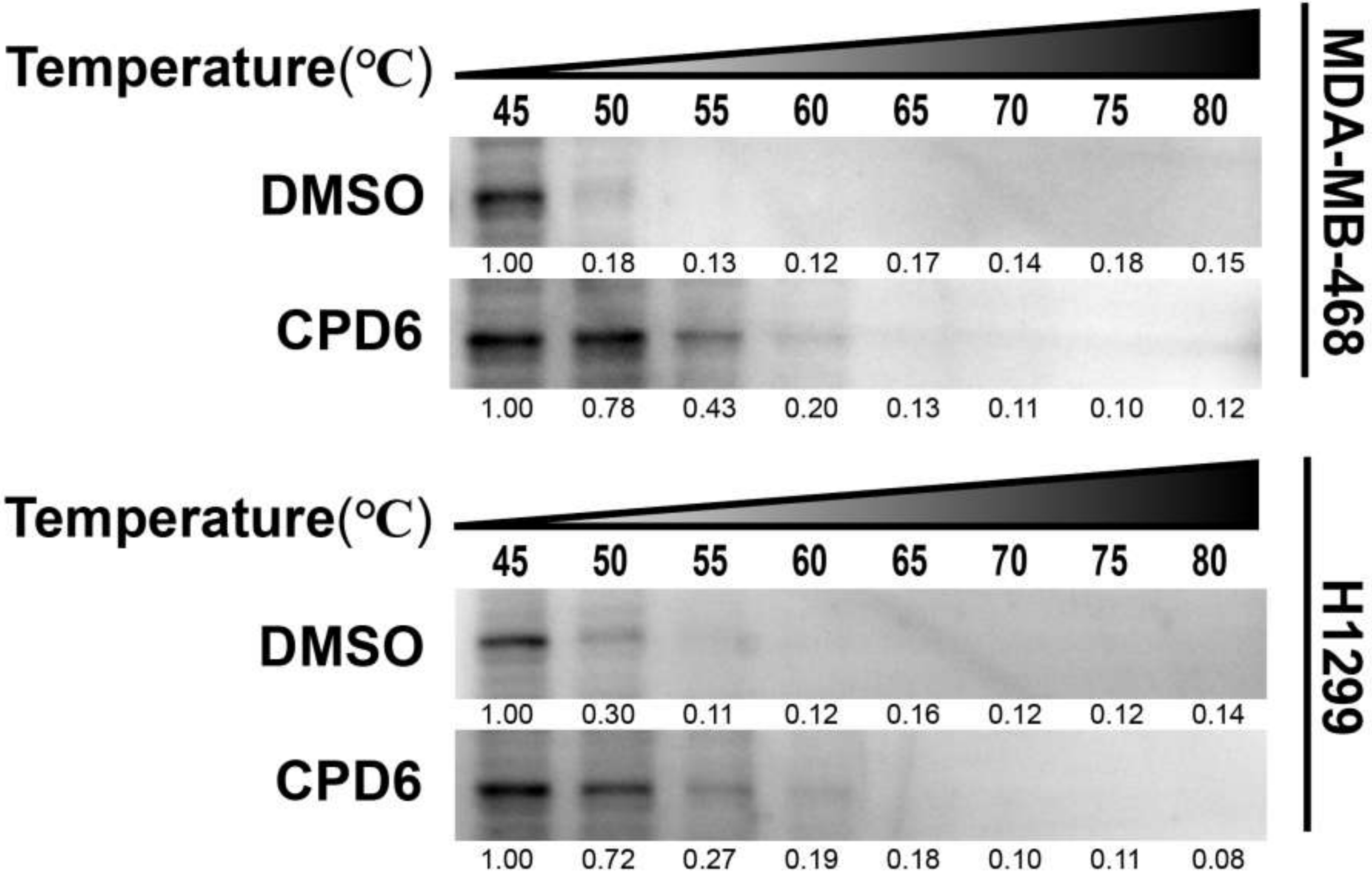

Temperature(°C)
45
50
55
60
65
70
75
80
MDA-MB-468
DMSO
1.00 0.18 0.13 0.12 0.17 0.14 0.18 0.15
CPD6
1.00 0.78 0.43 0.20 0.13 0.11 0.10 0.12
Temperature(°C)
45
50
55
60
65
70
75
80
H1299
DMSO
1.00 0.30 0.11 0.12 0.16 0.12 0.12 0.14
CPD6
1.00 0.72 0.27 0.19 0.18 0.10 0.11 0.08

Cell type:MDA-MB-468
Target protein:MELK
Sample ID:60
Corresponds to main text Figure 5C.
Red-framed bands are shown in the main text figure.

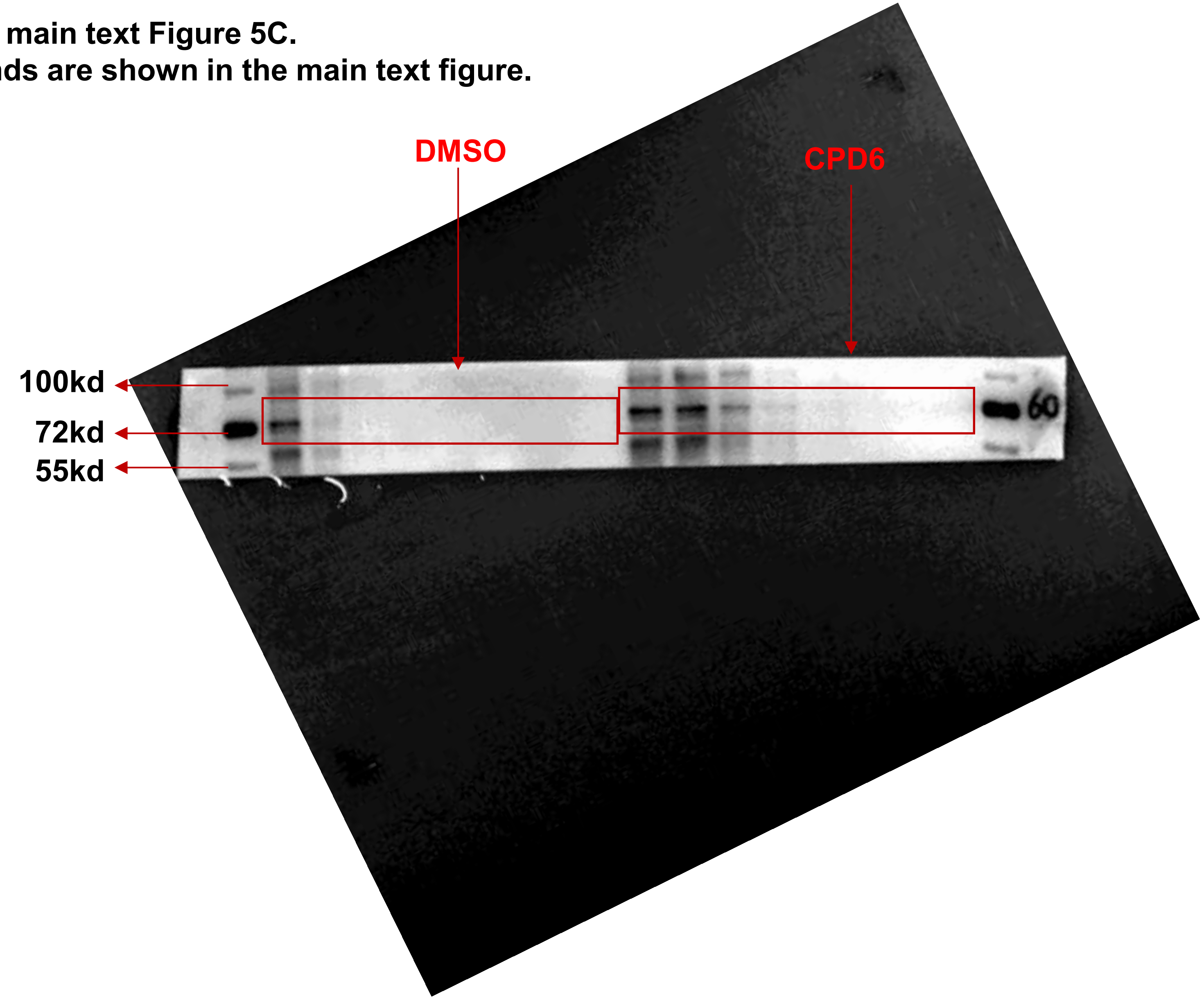

Cell type:H1299
Target protein:MELK
Sample ID:57
Corresponds to main text Figure 5C.
Red-framed bands are shown in the main text figure.

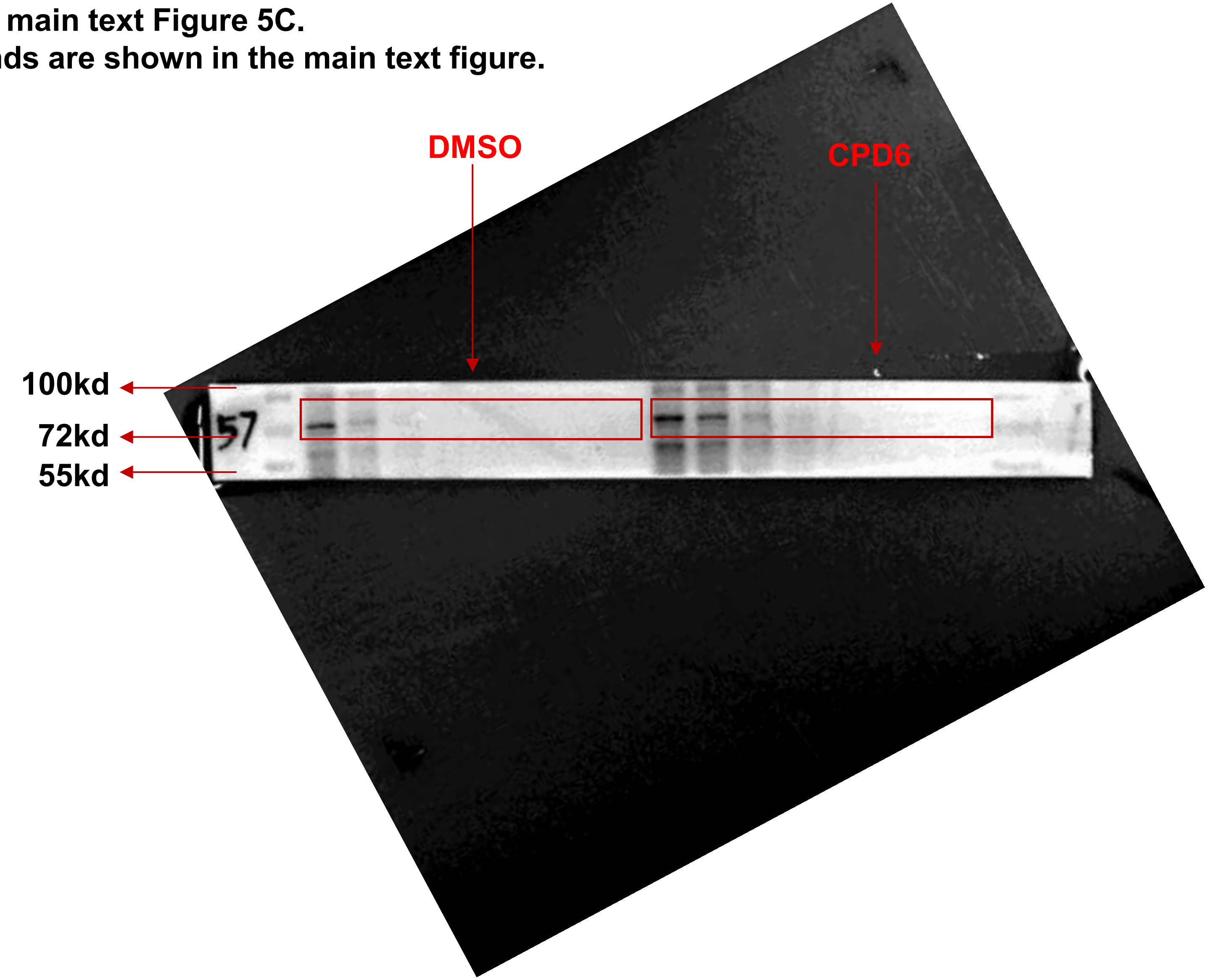

Fig 5D

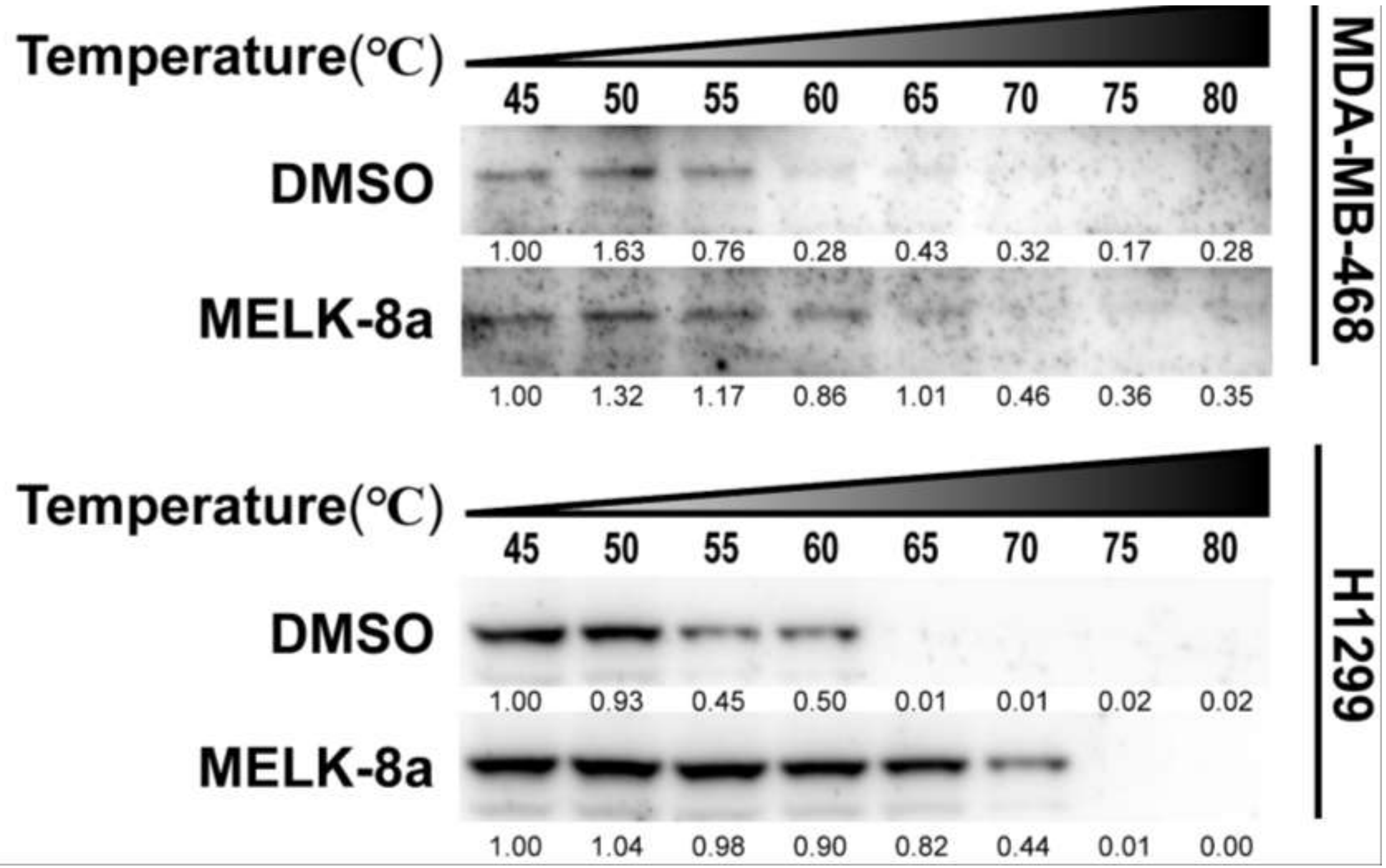
MDA-MB-468
Temperature(°C)
45 50 55 60 65 70 75 80
DMSO
1.00 1.63 0.76 0.28 0.43 0.32 0.17 0.28
MELK-8a
1.00 1.32 1.17 0.86 1.01 0.46 0.36 0.35
H1299
Temperature(°C)
45 50 55 60 65 70 75 80
DMSO
1.00 0.93 0.45 0.50 0.01 0.01 0.02 0.02
MELK-8a
1.00 1.04 0.98 0.90 0.82 0.44 0.01 0.00

Cell type:MDA-MB-468
Target protein:MELK
Sample ID:13
Corresponds to main text Figure 5D.
Red-framed bands are shown in the main text figure.

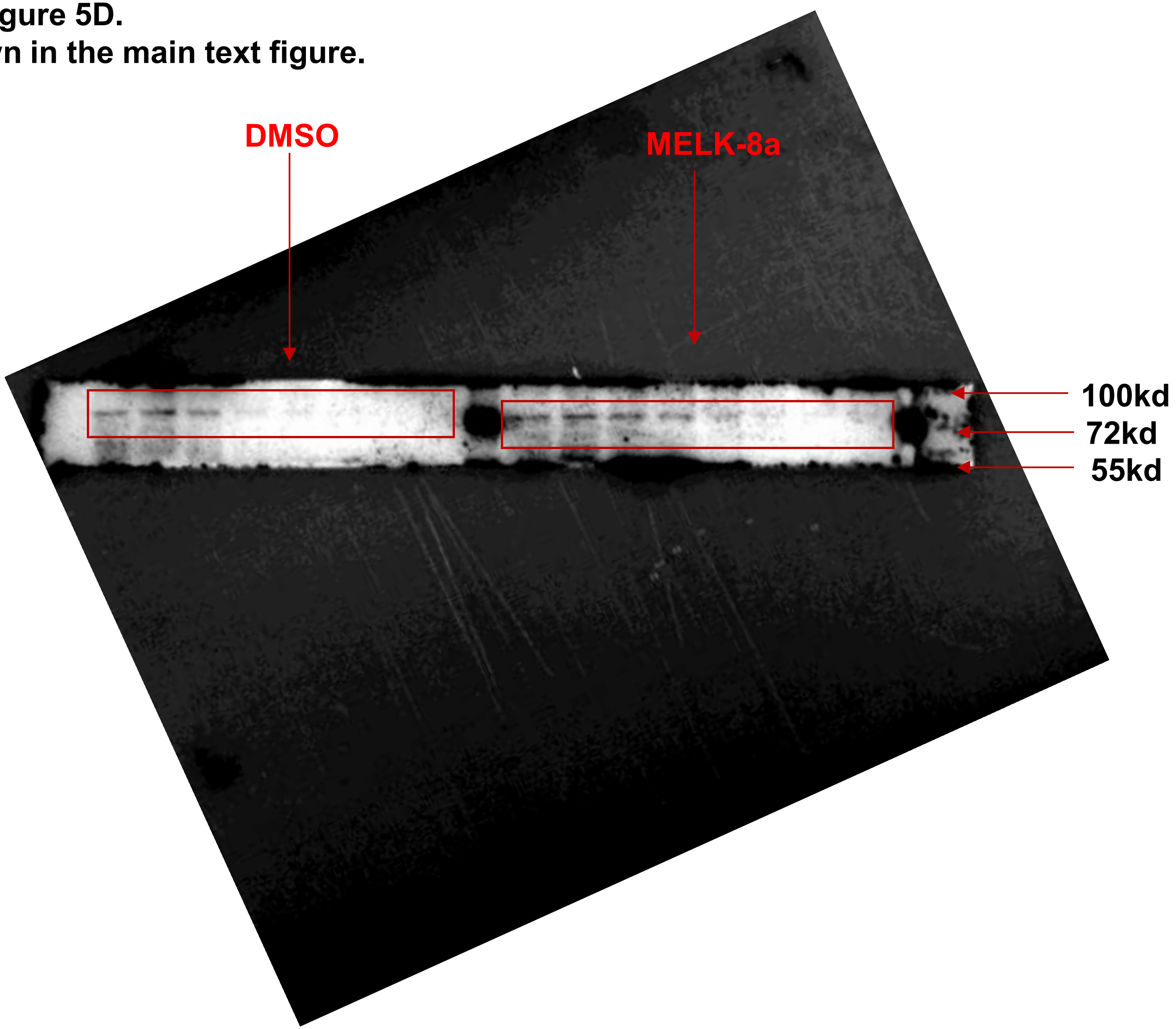

Cell type:H1299
Target protein:MELK
Sample ID:14
Corresponds to main text Figure 5D.
Red-framed bands are shown in the main text figure.

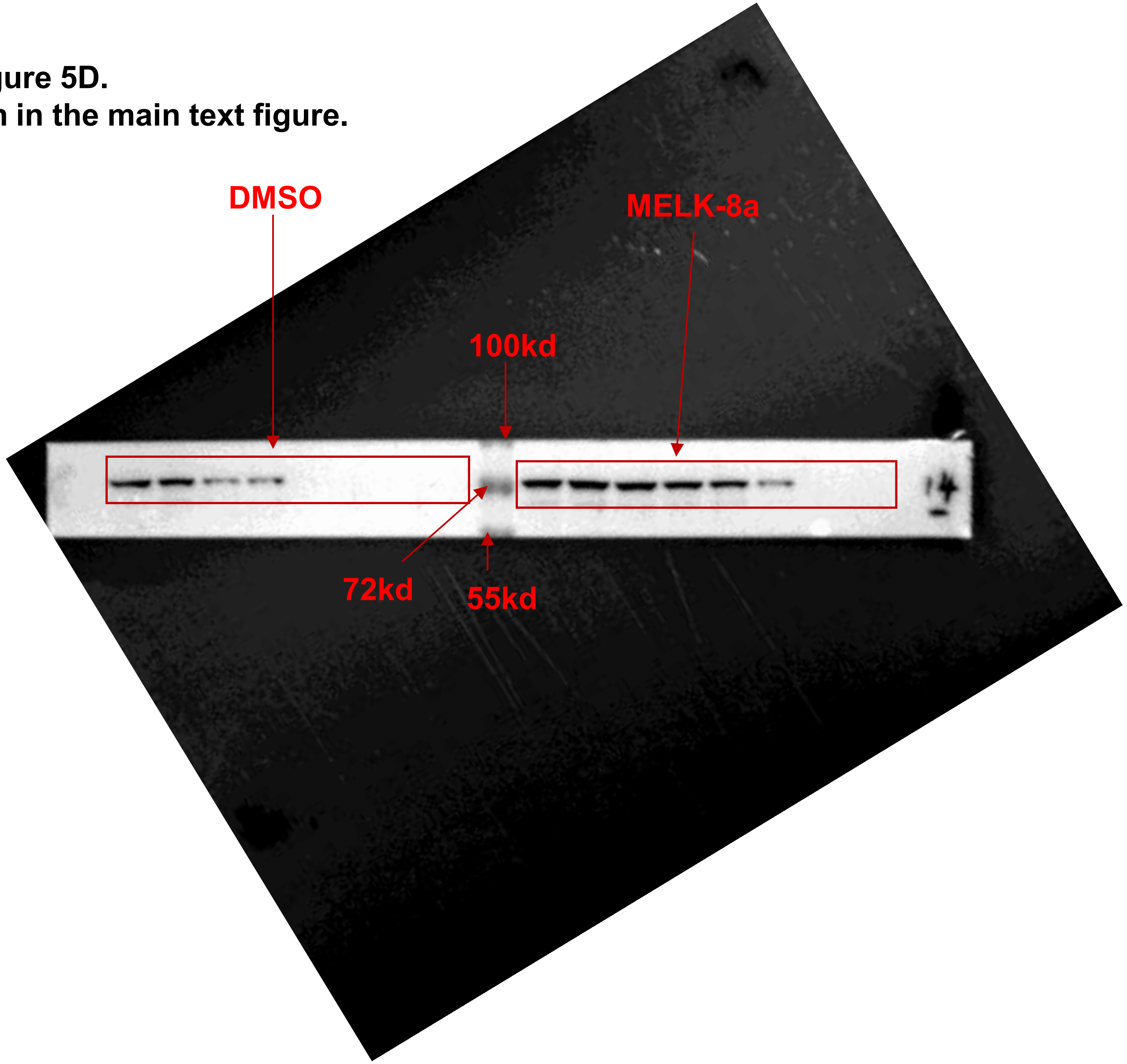

Fig 5E

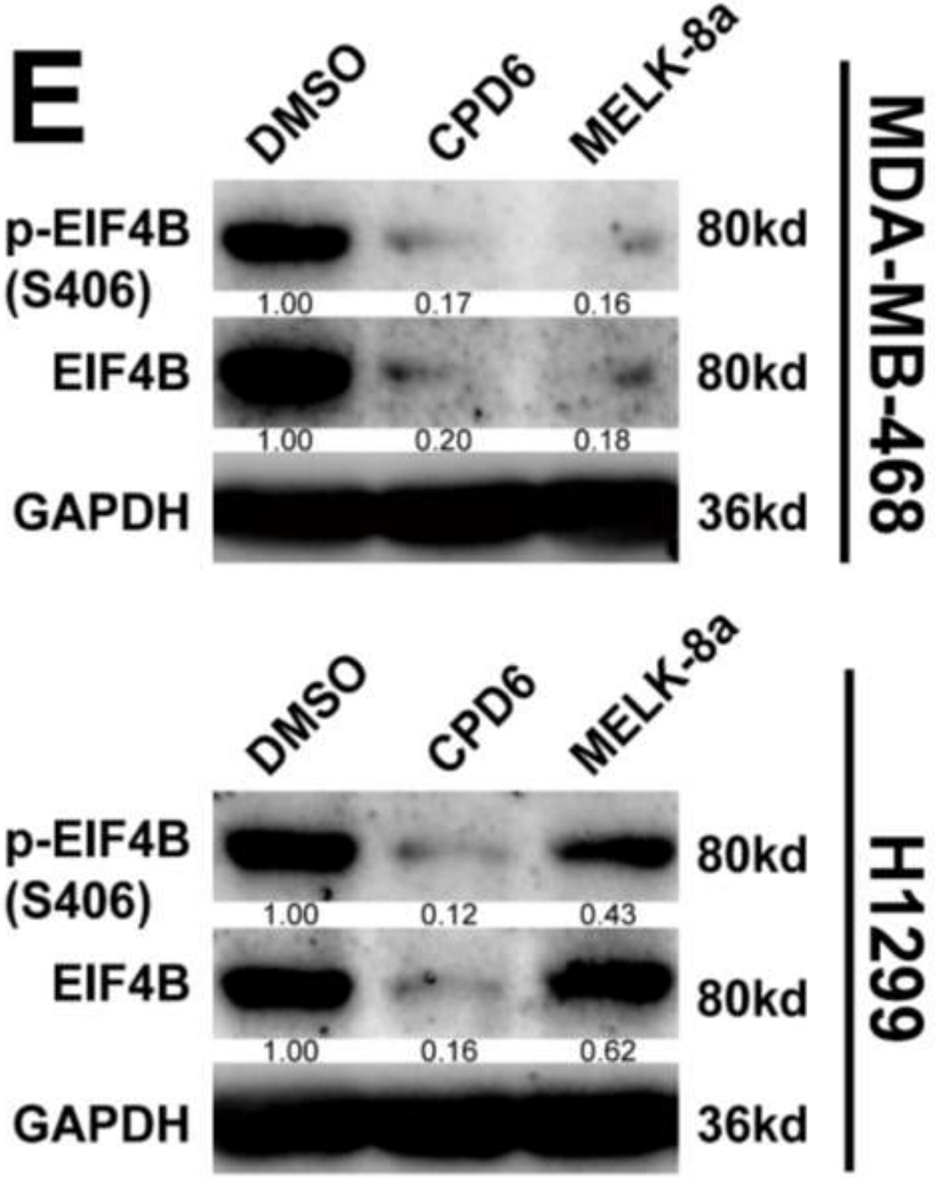
E
DMSO
CPD6
MELK-8a
p-EIF4B (S406)
80kd
1.00
0.17
0.16
EIF4B
80kd
1.00
0.20
0.18
GAPDH
36kd
MDA-MB-468
DMSO
CPD6
MELK-8a
p-EIF4B (S406)
80kd
1.00
0.12
0.43
EIF4B
80kd
1.00
0.16
0.62
GAPDH
36kd
H1299

Cell type:MDA-MB-468
Target protein:p-EIF4B(S406)
Sample ID:17
Corresponds to main text Figure 5E.
Red-framed bands are shown in the main text figure.

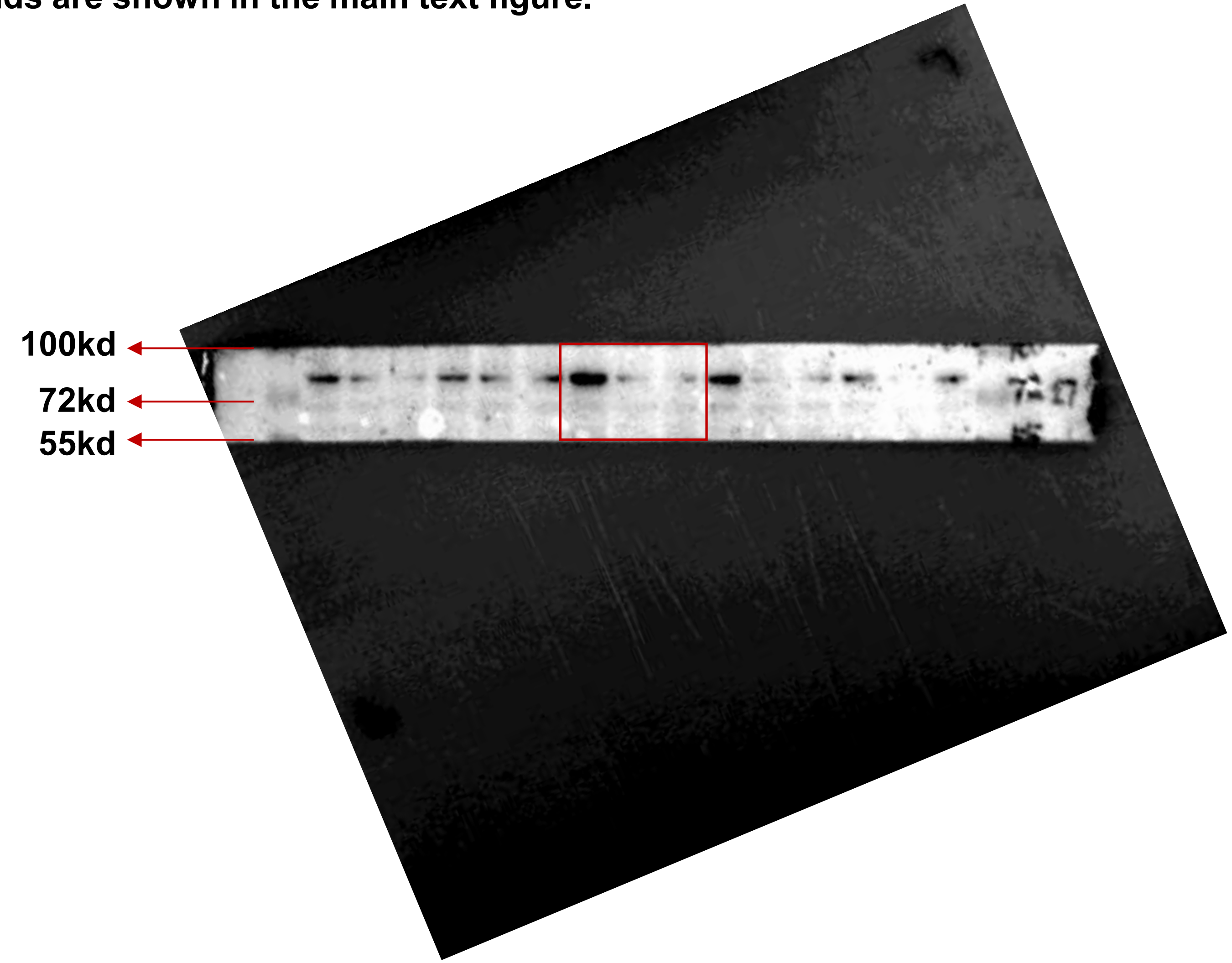

Cell type:MDA-MB-468
Target protein:EIF4B
Sample ID:17
Corresponds to main text Figure 5E.
Red-framed bands are shown in the main text figure.

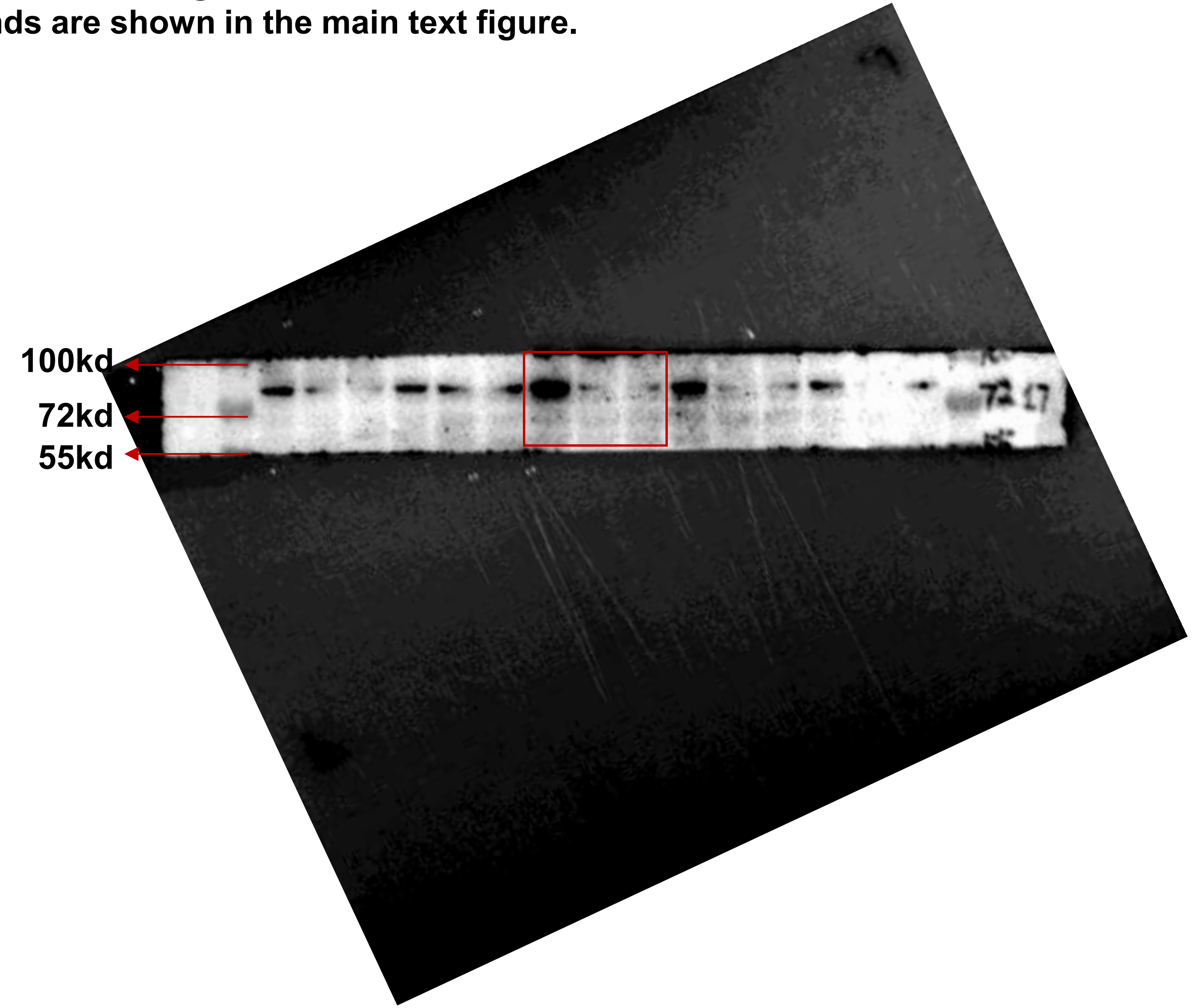

Cell type:MDA-MB-468
Target protein:gapdh
Sample ID:17
Corresponds to main text Figure 5E.
Red-framed bands are shown in the main text figure.

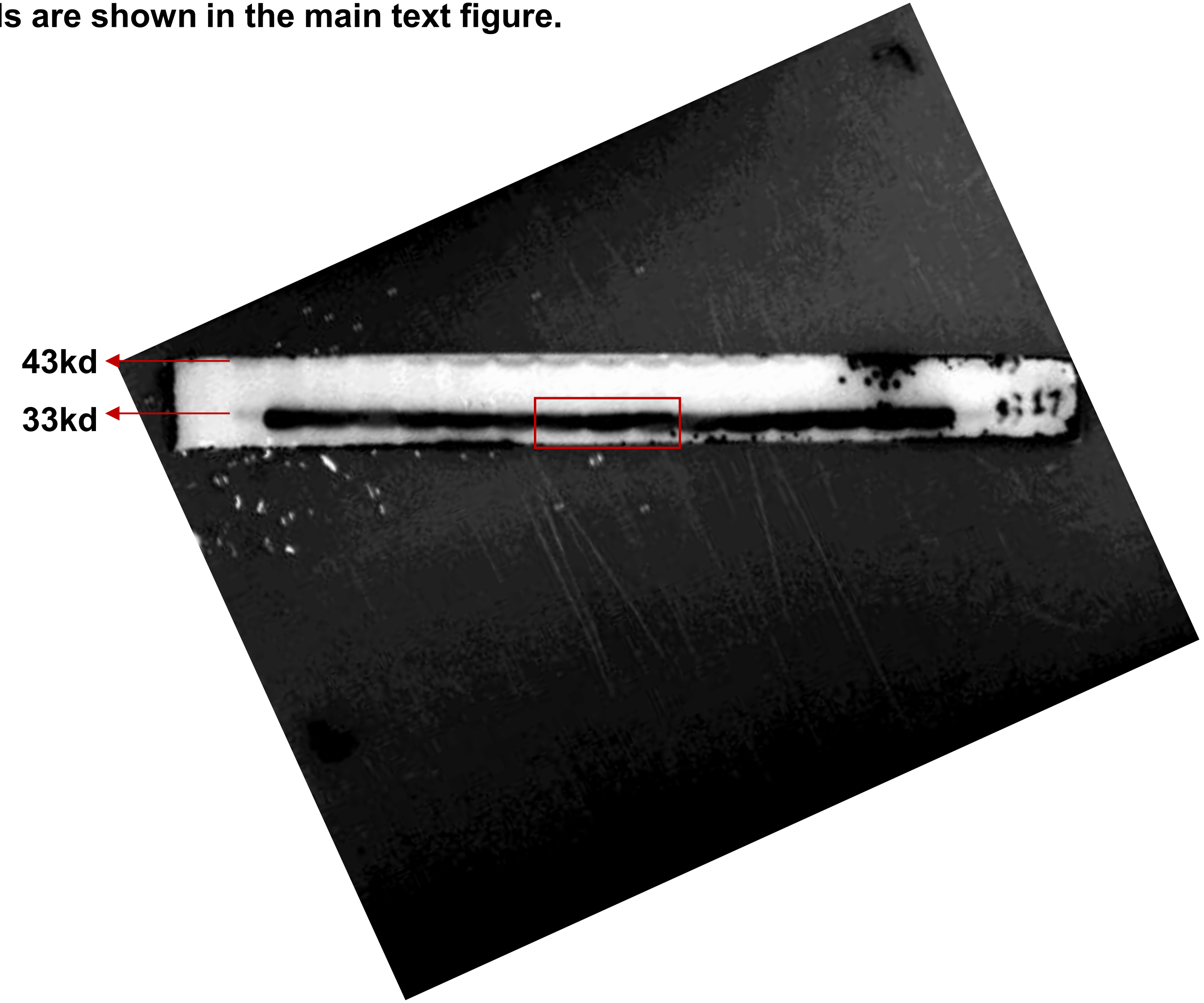

Cell type:H1299
Target protein:p-EIF4B(S406)
Sample ID:19
Corresponds to main text Figure 5E.
Red-framed bands are shown in the main text figure.

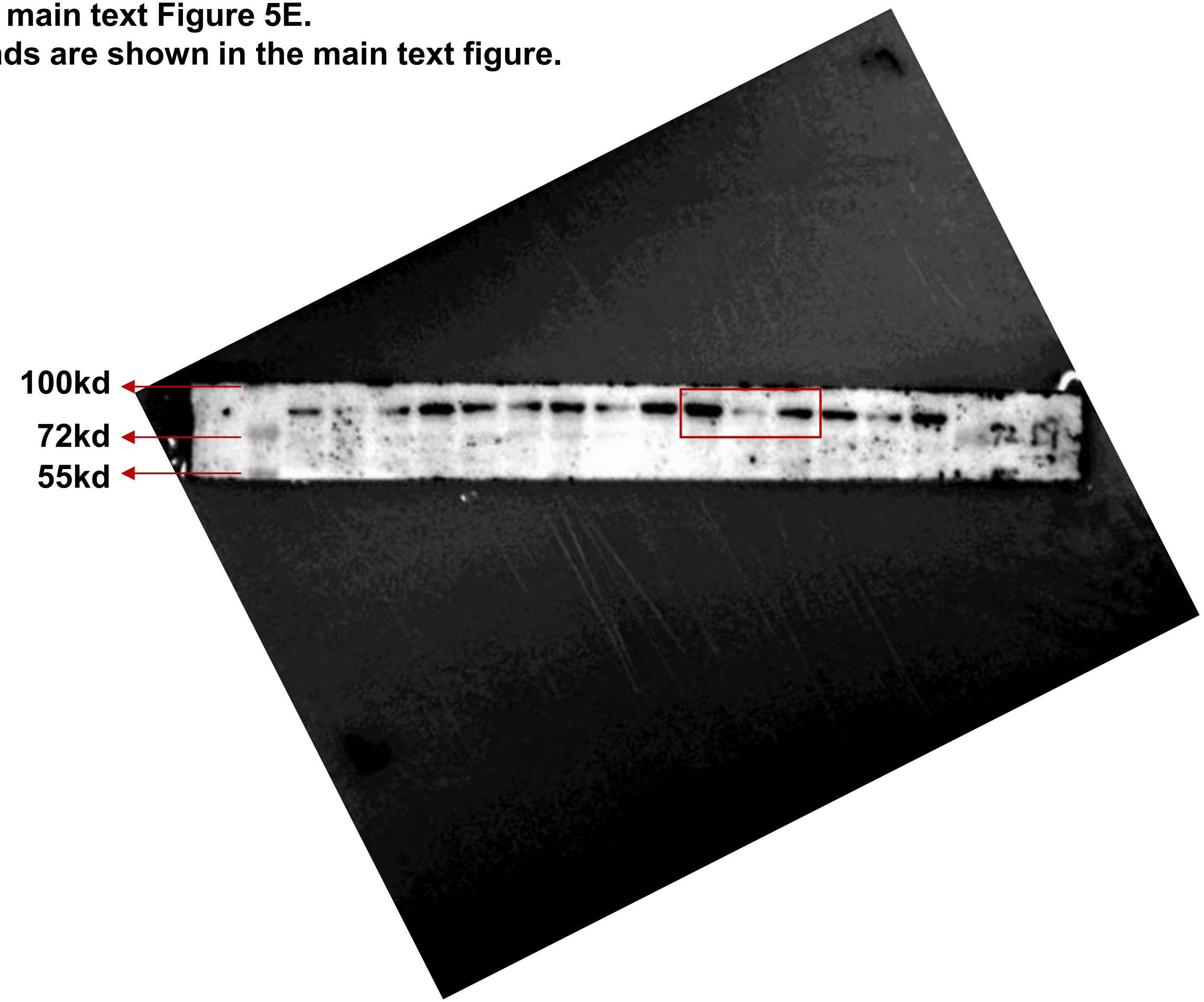

Cell type:H1299
Target protein:EIF4B
Sample ID:19
Corresponds to main text Figure 5E.
Red-framed bands are shown in the main text figure.

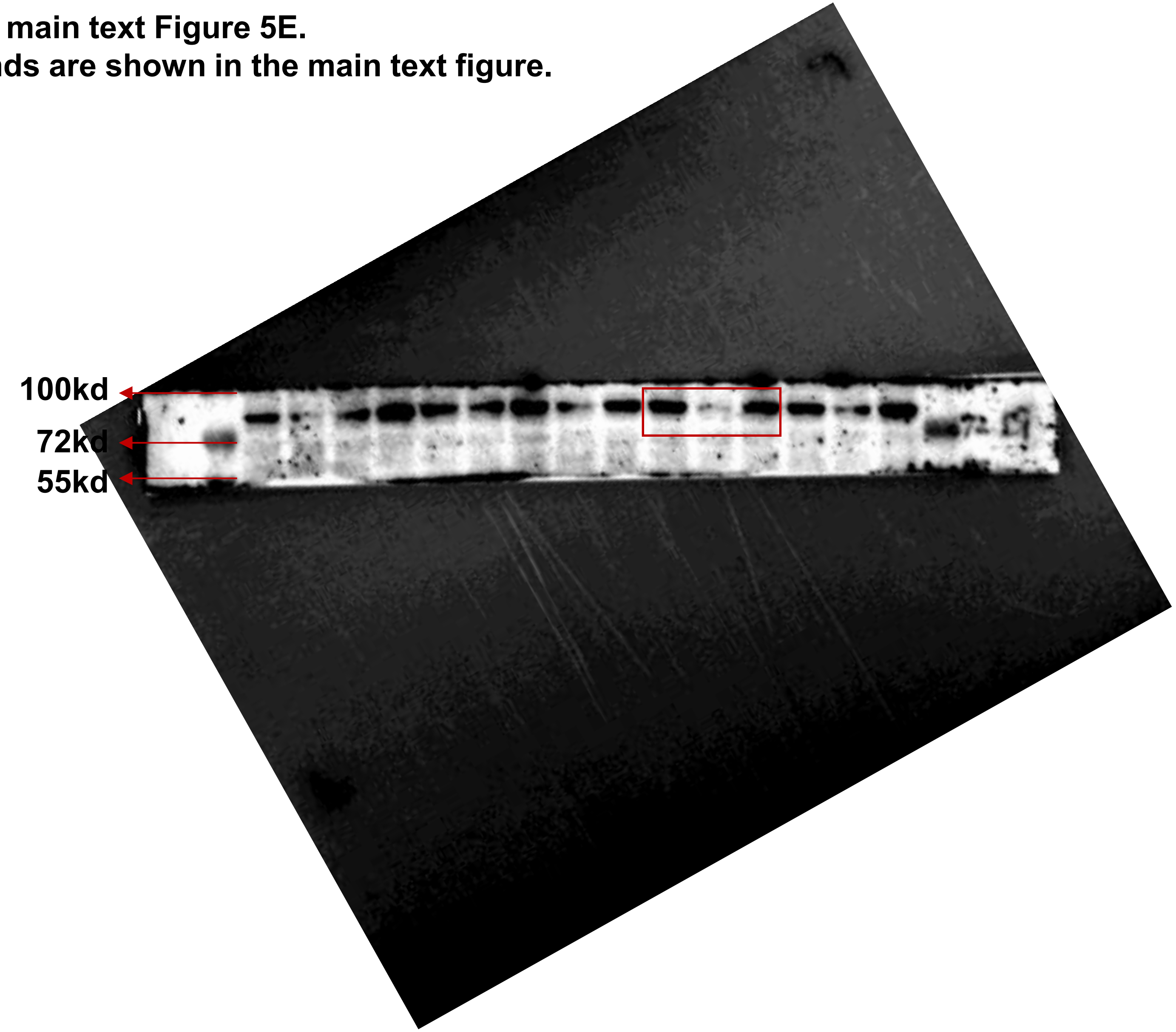

Cell type:H1299
Target protein:gapdh
Sample ID:19
Corresponds to main text Figure 5E.
Red-framed bands are shown in the main text figure.

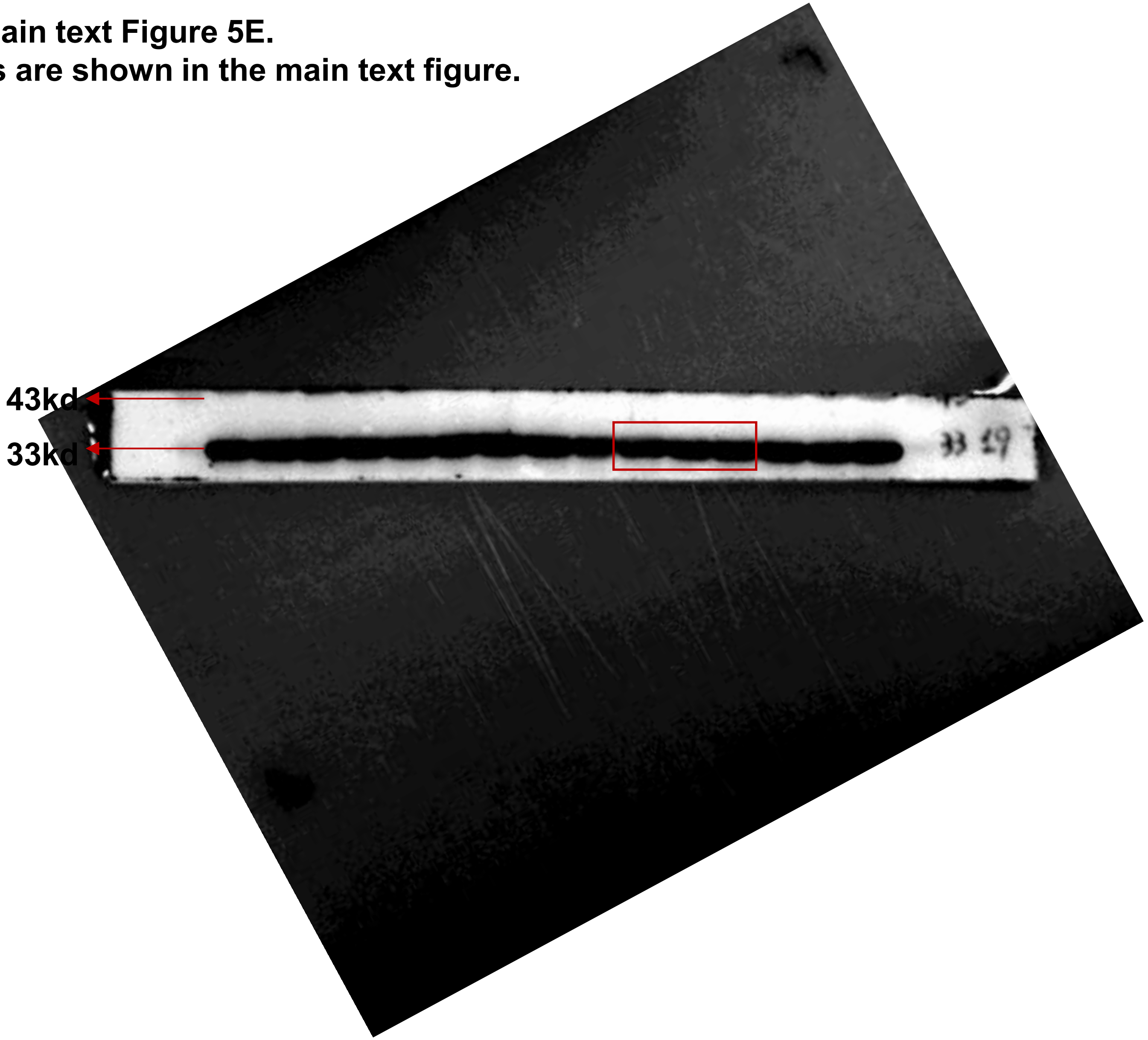

Fig 7C

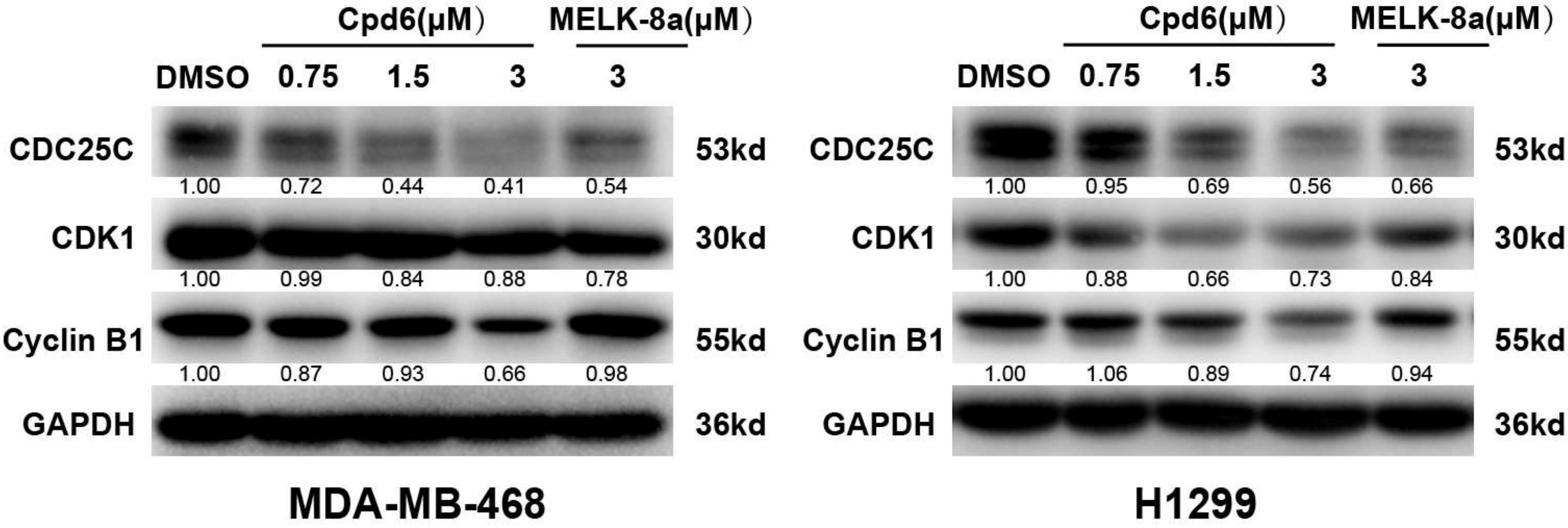

Cpd6(μM)
MELK-8a(μM)
DMSO
0.75
1.5
3
3
CDC25C
53kd
1.00 0.72 0.44 0.41 0.54
CDK1
30kd
1.00 0.99 0.84 0.88 0.78
Cyclin B1
55kd
1.00 0.87 0.93 0.66 0.98
GAPDH
36kd
MDA-MB-468
Cpd6(μM)
MELK-8a(μM)
DMSO
0.75
1.5
3
3
CDC25C
53kd
1.00 0.95 0.69 0.56 0.66
CDK1
30kd
1.00 0.88 0.66 0.73 0.84
Cyclin B1
55kd
1.00 1.06 0.89 0.74 0.94
GAPDH
36kd
H1299

Cell type:MDA-MB-468
Target protein:CDC25C
Sample ID:67
Corresponds to main text Figure 7C.
Red-framed bands are shown in the main text figure.

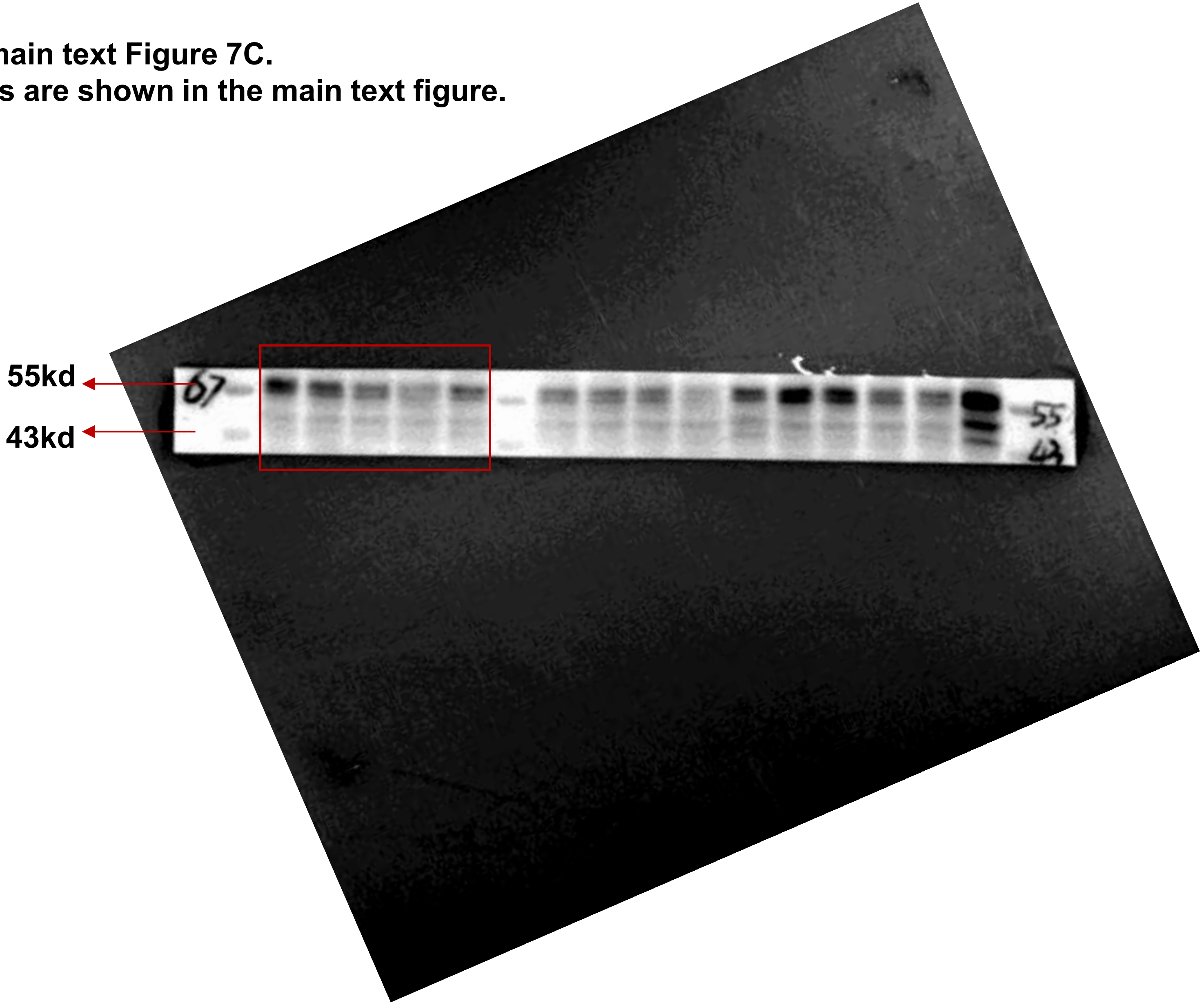

Cell type:MDA-MB-468
Target protein:CDK1
Sample ID:67
Corresponds to main text Figure 7C.
Red-framed bands are shown in the main text figure.

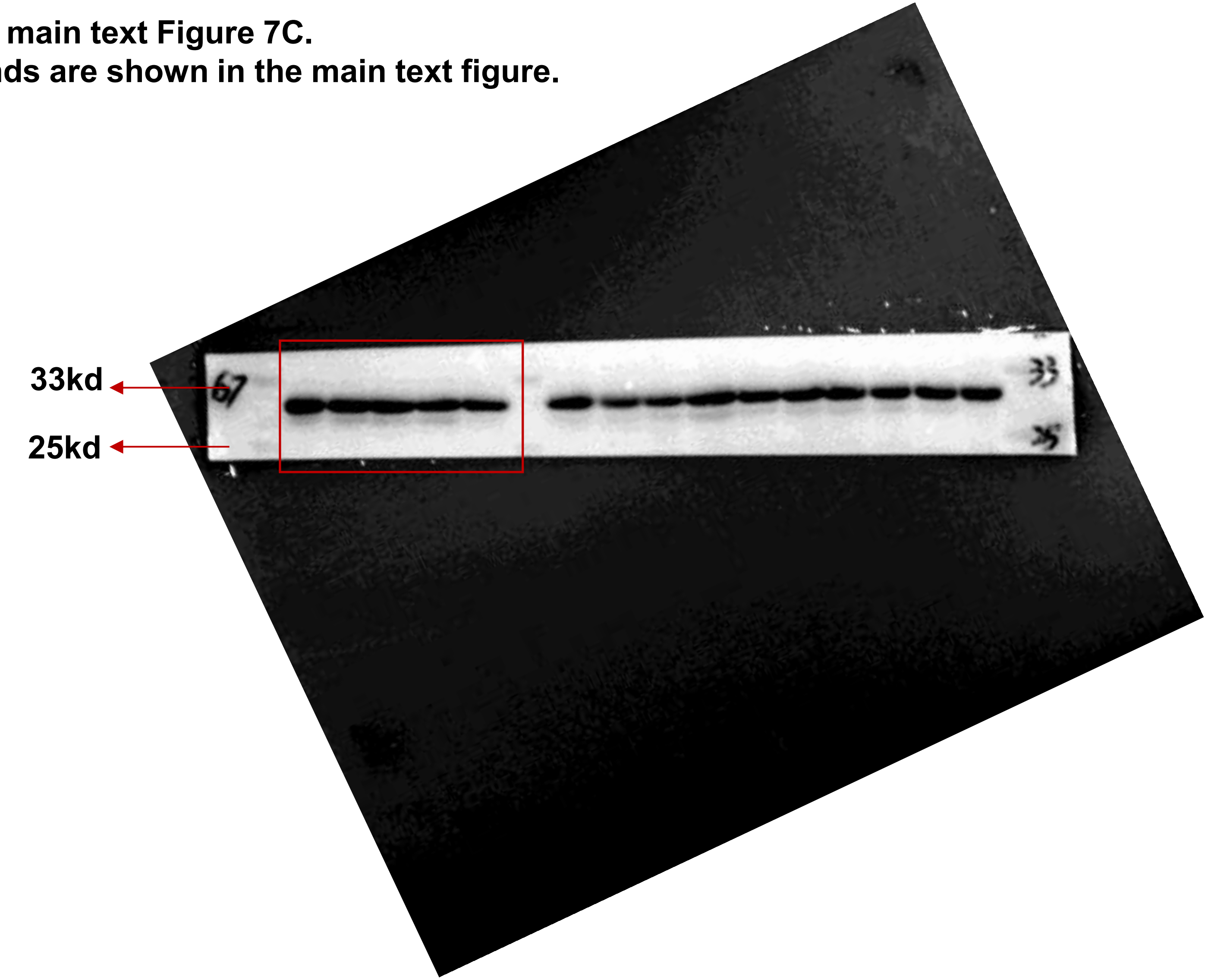

Cell type:MDA-MB-468
Target protein:gapdh
Sample ID:67
Corresponds to main text Figure 7C.
Red-framed bands are shown in the main text figure.

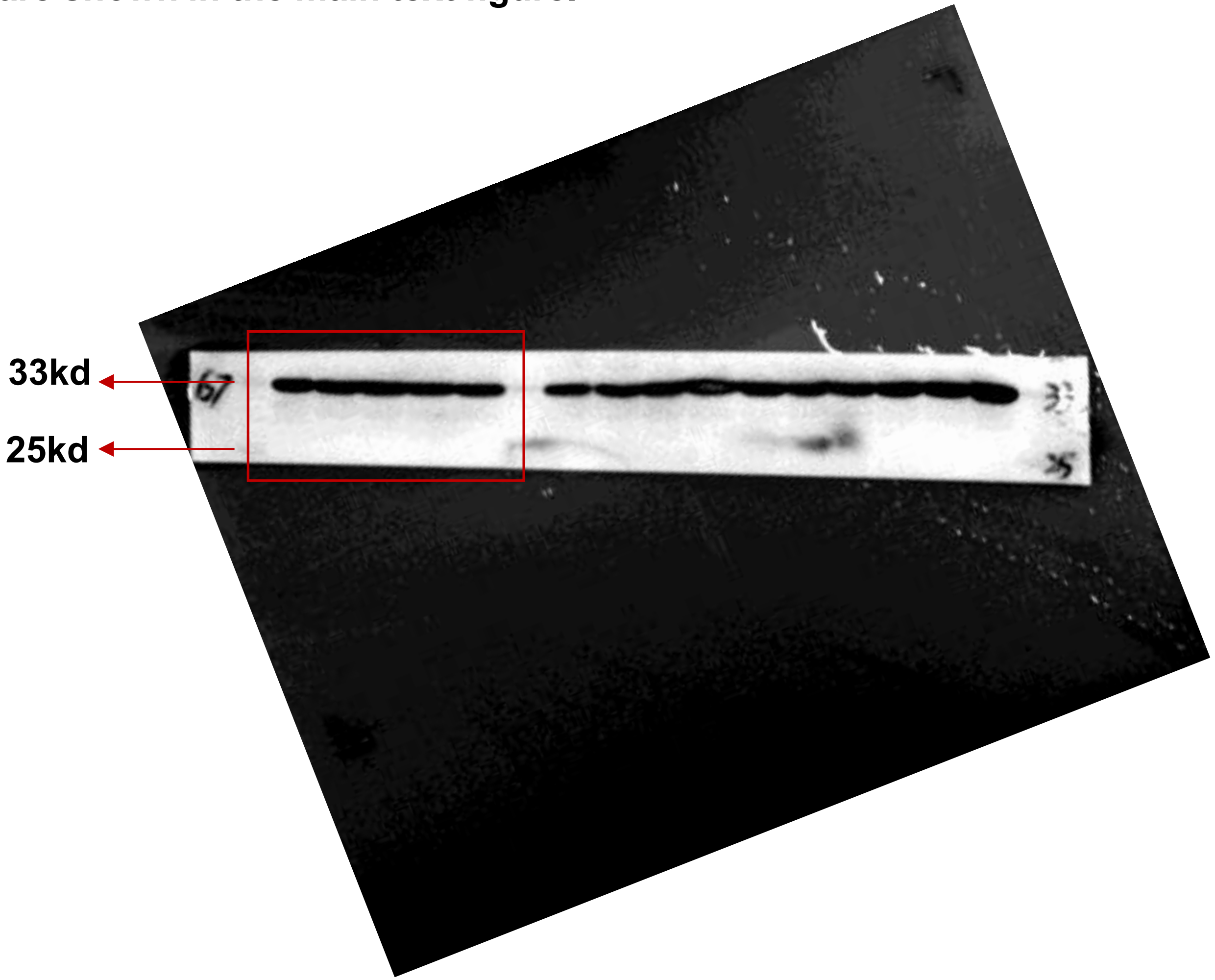

Cell type:MDA-MB-468
Target protein:Cyclin B1
Sample ID:45
Corresponds to main text Figure 7C.
Red-framed bands are shown in the main text figure.

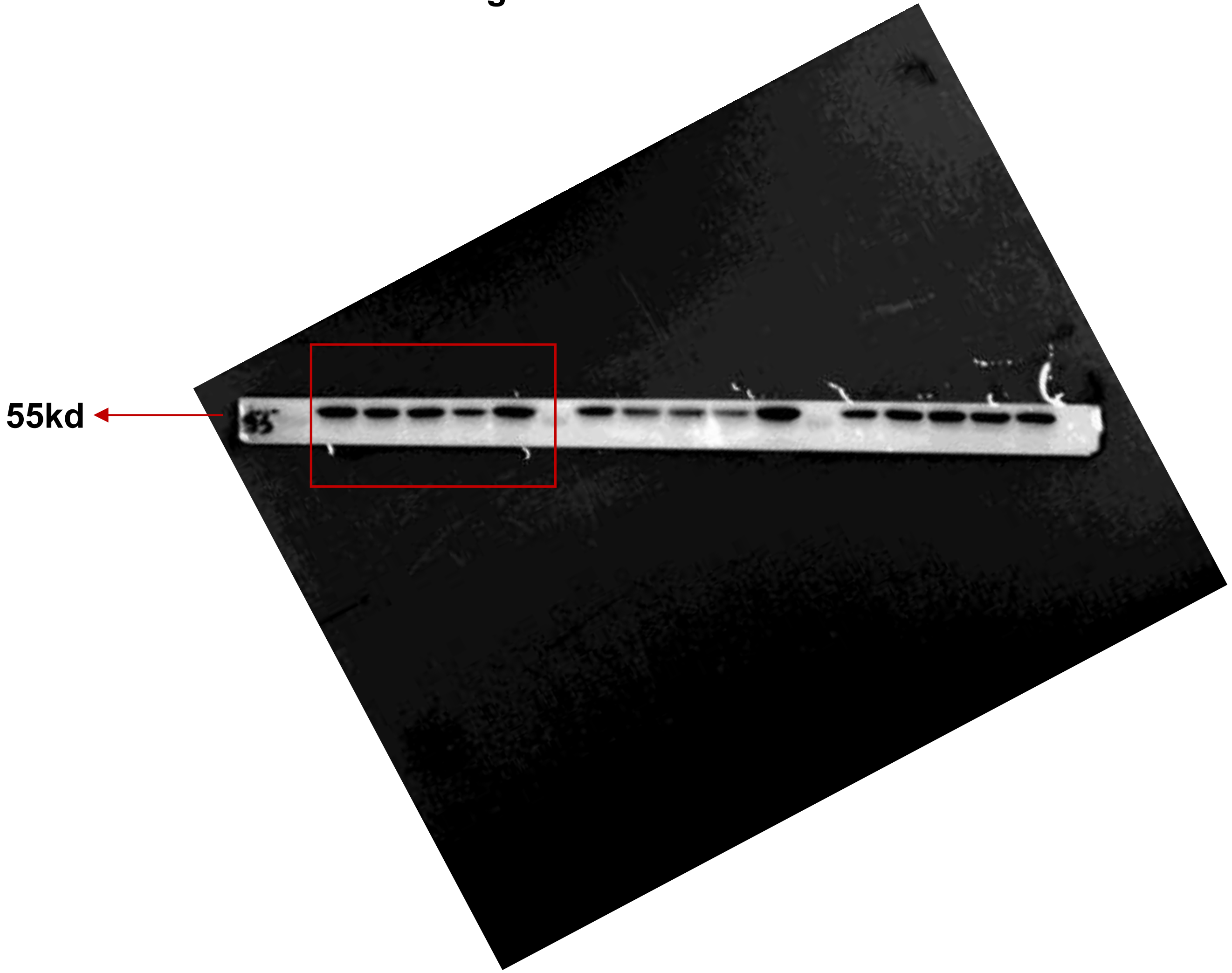

Cell type:MDA-MB-468
Target protein:gapdh
Sample ID:45
Corresponds to main text Figure 7C.
Red-framed bands are shown in the main text figure.

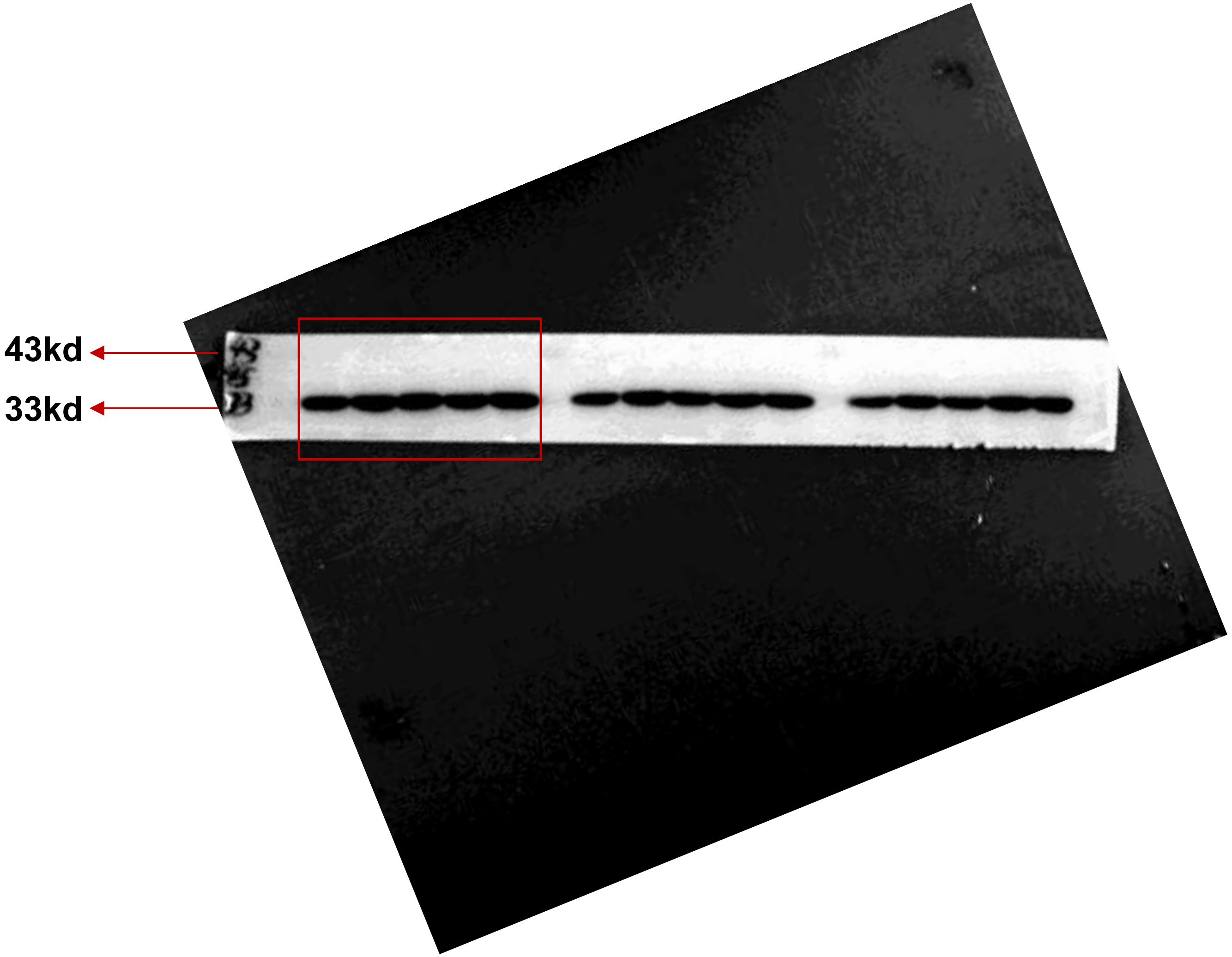

Cell type:H1299
Target protein:CDC25C
Sample ID:66
Corresponds to main text Figure 7C.
Red-framed bands are shown in the main text figure.

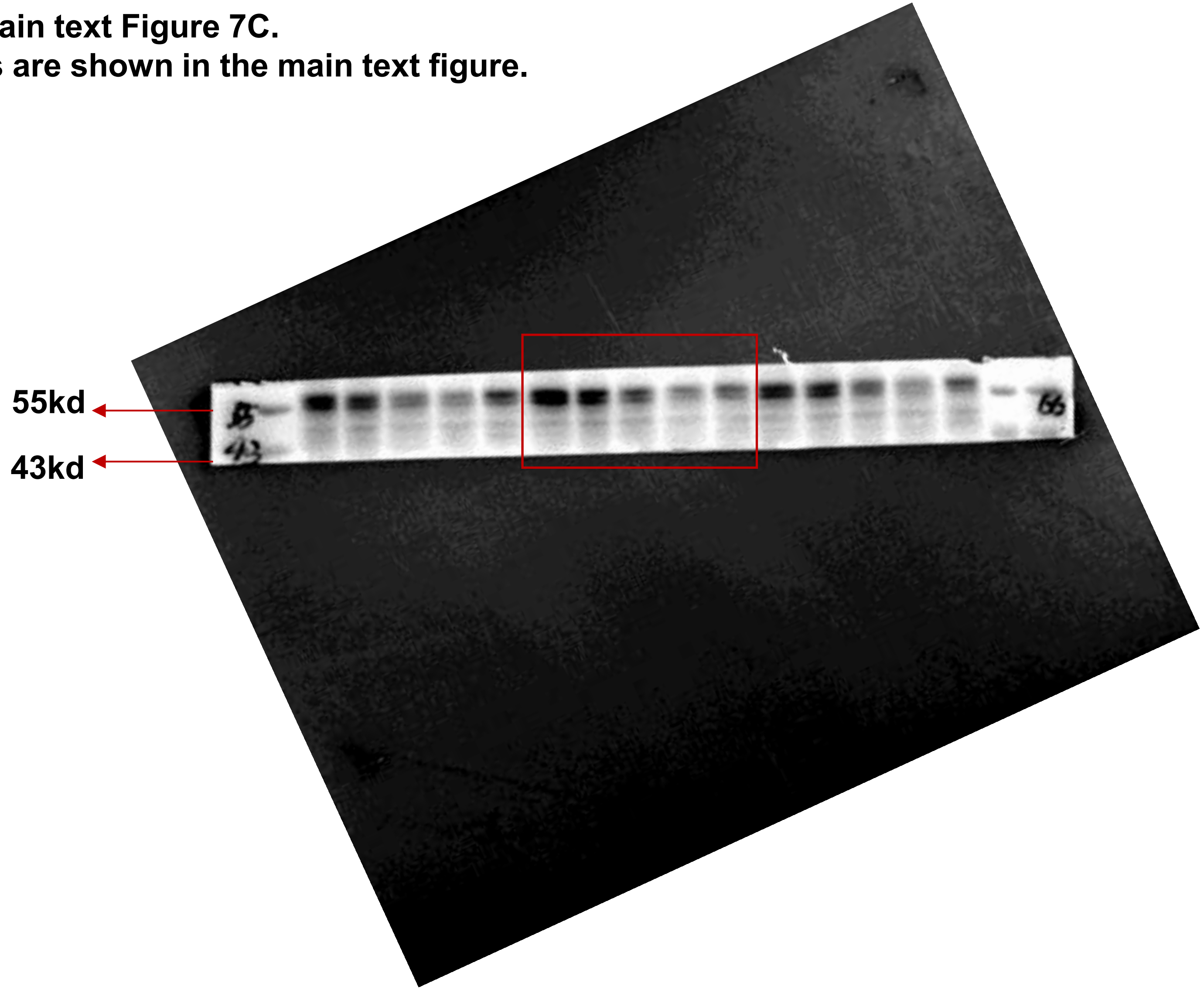

Cell type:H1299
Target protein:CDK1
Sample ID:66
Corresponds to main text Figure 7C.
Red-framed bands are shown in the main text figure.

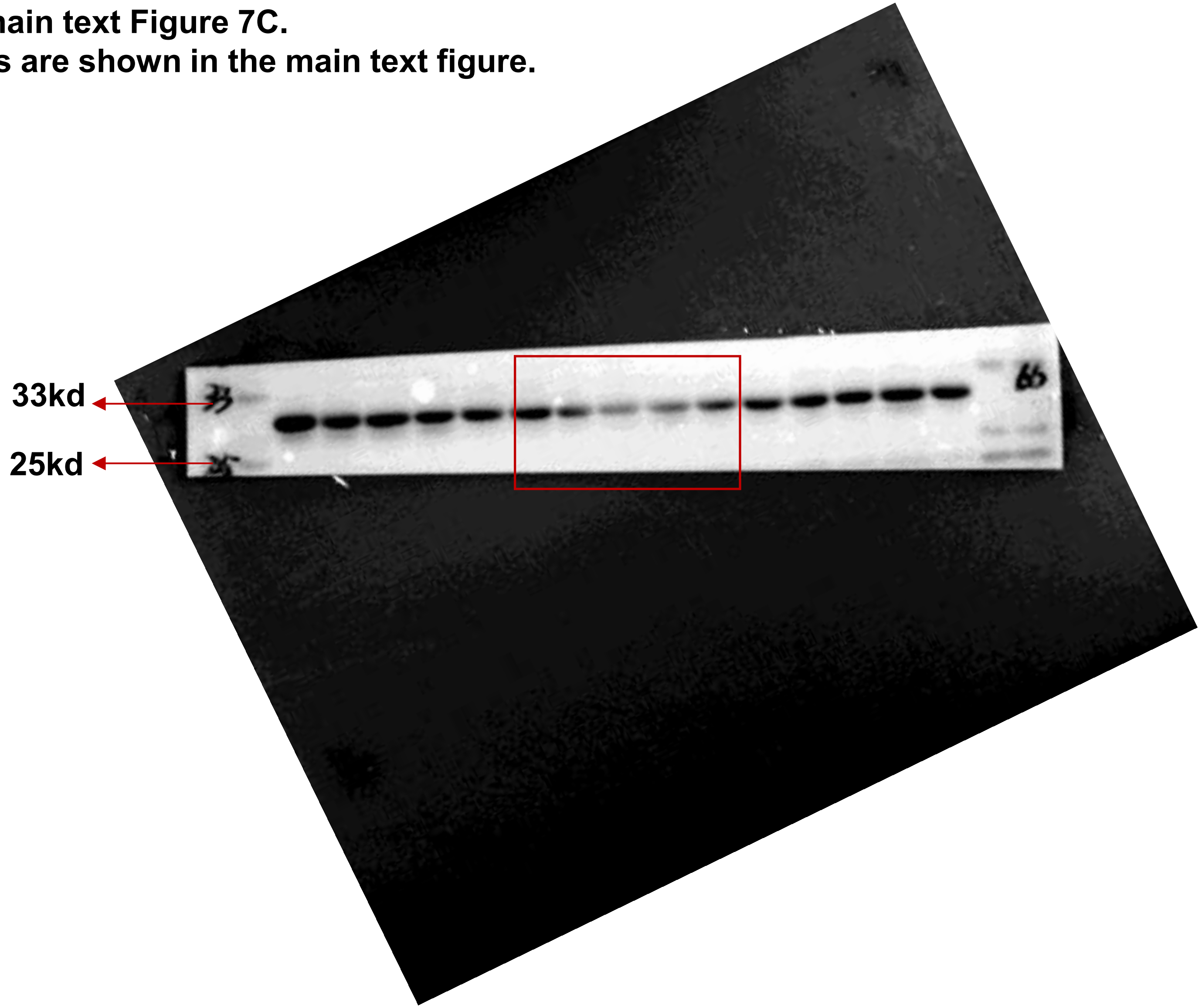

Cell type:H1299
Target protein:Cyclin B1
Sample ID:66
Corresponds to main text Figure 7C.
Red-framed bands are shown in the main text figure.

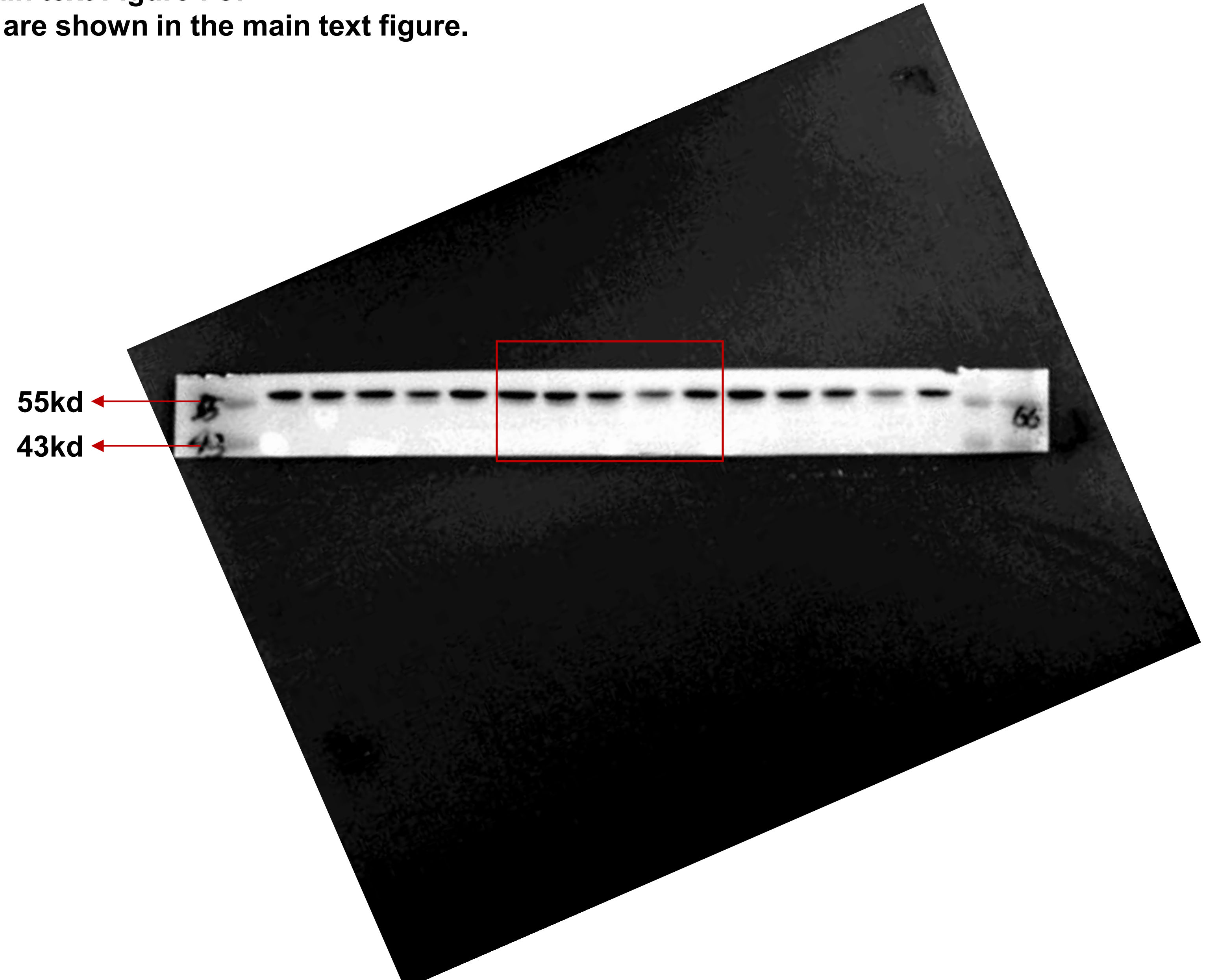

Cell type:H1299
Target protein:gapdh
Sample ID:66
Corresponds to main text Figure 7C.
Red-framed bands are shown in the main text figure.

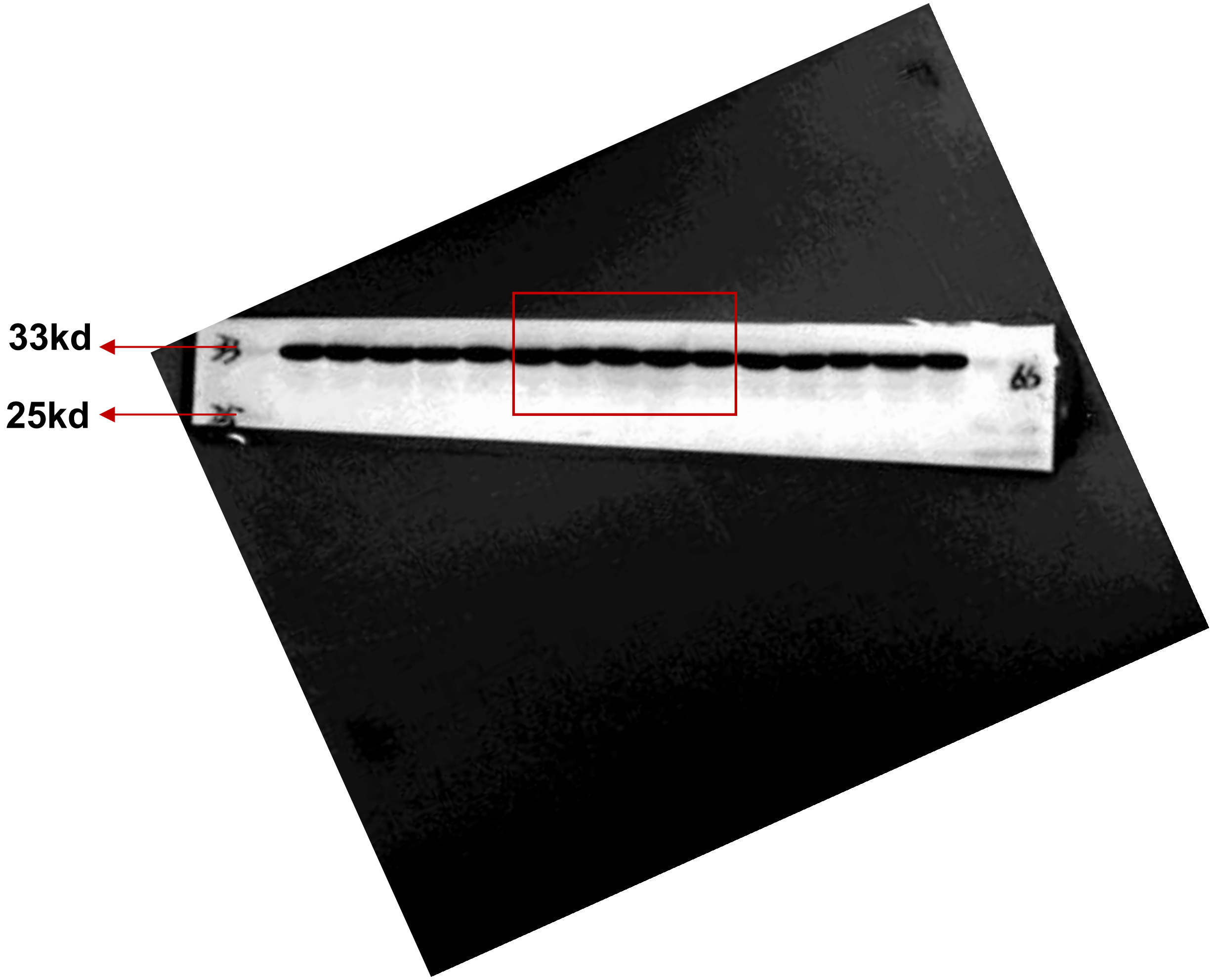

Supplement: Supplementary file 1 [file cancers-18-03336-s001.zip › cancers-4571407-supplementary/Supplementary Files/cancers-4571407-File S1-original western blots.pdf]
