# Supplementary material for: Machine Learning-Guided Virtual Screening Identifies a Novel MELK-Targeted Antiproliferative Lead Compound with G2/M Arrest Activity in Triple-Negative Breast Cancer and Lung Adenocarcinoma Cells
Source: Cancers (Basel). 2026 Oct 15;18(20):3336. doi: 10.3390/cancers18203336 (PMC13702308; doi:10.3390/cancers18203336)
Supplement: Supplementary file 1 [file cancers-18-03336-s001.zip › cancers-4571407-supplementary/Supplementary Files/cancers-4571407-supplementary tables.pdf]

Supporting Information

Machine-Learning-Guided Virtual Screening Identifies a Novel MELK-Targeted Antiproliferative Lead Compound with G2/M Arrest Activity in Triple-Negative Breast Cancer and Lung Adenocarcinoma Cells

Table S1. Information of antibodies.

Antibody	Catalogue	Dilution	Company
GAPDH	10494-1-AP	1:1000	Proteintech, Europe
MELK	F2906	1:1000	Selleck, USA
CDC2	9116	1:1000	Cell Signaling Technology, USA
CDC25C	4688	1:1000	Cell Signaling Technology, USA
Cyclin B1	F2522	1:1000	Selleck, USA
p-EIF4B	F1305	1:1000	Selleck, USA
EIF4B	F2099	1:1000	Selleck, USA

Table S2. Performance Metrics of Various Machine Learning Models Evaluated by 10-Fold Cross-Validation.

10-Fold	R^2	MSE	RMSE	MAE
RF	0.7566 ± 0.0610	0.2647 ± 0.0733	0.5099 ± 0.0684	0.3569 ± 0.0316
GBR	0.7586 ± 0.0664	0.2626 ± 0.0786	0.5070 ± 0.0745	0.3420 ± 0.0421
LightGBM	0.7067 ± 0.0837	0.3178 ± 0.0942	0.5575 ± 0.0833	0.3840 ± 0.0522
XGBoost	0.7454 ± 0.0703	0.2765 ± 0.0822	0.5207 ± 0.0733	0.3570 ± 0.0410
Ridge	0.7509 ± 0.0385	0.2696 ± 0.0404	0.5177 ± 0.0393	0.3497 ± 0.0206
SVR	0.7088 ± 0.0547	0.3163 ± 0.0675	0.5596 ± 0.0562	0.3732 ± 0.0319
PLS	0.6729 ± 0.0446	0.3551 ± 0.0568	0.5941 ± 0.0464	0.4212 ± 0.0312
KNN	0.6434 ± 0.0912	0.3858 ± 0.1025	0.6161 ± 0.0788	0.4194 ± 0.0460
MLP	0.6970 ± 0.0684	0.3275 ± 0.0748	0.5688 ± 0.0635	0.3801 ± 0.0416
DTR	0.5773 ± 0.1114	0.4529 ± 0.1022	0.6680 ± 0.0819	0.4478 ± 0.0455
ElasticNet	0.4986 ± 0.0667	0.5440 ± 0.0803	0.7356 ± 0.0540	0.5409 ± 0.0355

Table S3. ADMET Prediction Results of 12 potential MELK compounds.

Compound ID	Absorption			Distribution			Metabolism		Excretion	Toxicity
	HIA	Caco-2	MDCK	BBB	PPB	VD	CYP2D6 sub	CYP3A4 sub	$T_{1/2}$	Ames
CPD1	0.001	-5.005	-4.650	0.006	98.900	0.456	0.000	0.992	0.568	0.781
CPD2	0.000	-5.430	-5.166	0.000	98.690	0.604	0.262	0.000	0.870	0.798
CPD3	0.000	-5.610	-5.101	0.000	74.446	0.953	0.750	0.167	0.965	0.664
CPD4	0.000	-5.406	-5.186	0.000	98.117	0.432	0.020	0.000	0.841	0.802
CPD5	0.002	-5.163	-4.856	0.342	93.549	0.763	0.057	0.170	0.744	0.590
CPD6	0.000	-5.286	-5.135	0.000	98.678	0.536	0.001	0.000	0.928	0.789

CPD7	0.083	-4.977	-4.643	0.079	97.406	0.750	0.078	0.202	0.706	0.577
CPD8	0.000	-5.389	-5.133	0.000	98.768	0.584	0.008	0.019	0.792	0.774
CPD9	0.000	-5.491	-5.154	0.000	99.042	0.530	0.027	0.000	0.920	0.695
CPD10	0.000	-5.689	-5.153	0.000	92.436	0.370	0.955	0.652	1.118	0.568
CPD11	0.000	-4.679	-4.542	0.036	96.976	0.715	0.000	0.000	0.604	0.787
CPD12	0.000	-4.860	-4.777	0.931	86.354	0.804	0.113	0.989	0.426	0.759

Table S4. Basic information of candidate compounds.

Compound ID	Database source	SMILES
MELK-8a [1]		CN1CCN(C2=CC=C(N3C=C(C(C4=CC=NC=C4OCC5CC[N]CC5) C=N3)C=C2)CC1
CPD1	ZINC	COc1cc(-c2cc(CCCC(=O)Nc3ccccc3[N+](=O)[O-])[nH]n2)ccc1O
CPD2	Chemdiv	CCOc1ccc(-c2cc(C(=O)N/N=C/c3cccc(OC)c3O)[nH]n2)cc1
CPD3	Chemdiv	COc1ccc(O)c(/C=N/NC(=O)CNC(=O)c2ccc(OC)c(OC)c2)c1
CPD4	Chemdiv	COc1ccc(-c2cc(C(=O)N/N=C/c3cccc(OC)c3O)[nH]n2)cc1
CPD5	ZINC	COC(=O)c1c(-c2ccc(C(=O)NCc3noc(C4CC4)n3)cc2)cnn1C
CPD6	Chemdiv	COc1cccc(/C=N/NC(=O)c2cc(-c3ccc(F)cc3)n[nH]2)c1O
CPD7	ZINC	COc1ccc(-c2n[nH]c(SCC(=O)Nc3ccccc3O)n2)cc1
CPD8	Chemdiv	CCOc1cccc(-c2cc(C(=O)N/N=C/c3cccc(OC)c3O)[nH]n2)c1
CPD9	Chemdiv	CCOc1ccccc1-c1cc(C(=O)N/N=C/c2cccc(OC)c2O)[nH]n1
CPD10	Chemdiv	COc1ccc(C(=O)NCC(=O)N/N=C/c2cccc(OC)c2O)cc1OC
CPD11	ZINC	O=C(NC[C@H]1CCCO1)c1ccc(-c2ccc(Oc3ccccc3)nn2)cc1
CPD12	ZINC	Cc1nc(-c2ccc(CNC(=O)COC[C@@H]3CCCCO3)cc2)no1

[1] Touré BB, Giraldes J, Smith T, et al. Toward the validation of maternal embryonic leucine zipper kinase: Discovery, optimization of highly potent and selective inhibitors, and preliminary biology insight [J]. J Med Chem, 2016, 59: 4711-4723. https://doi.org/10.1021/acs.jmedchem.6b00052

Table S5. Predicted pIC50 values, docking scores, and MMGBSA binding energy calculations for 13 compounds.

Compound ID	RF	GBR	LightGBM	XGBoost	Docking score (kcal/mol)	ΔG_bind(kcal/mol)
MELK-8a	7.657	7.674	7.566	7.778	-9.49	-77.11
CPD1	7.002	7.328	6.756	7.079	-11.04	-84.65
CPD2	7.169	6.933	7.598	7.144	-11.97	-83.97
CPD3	7.026	7.116	6.902	7.159	-10.36	-83.96
CPD4	7.161	6.932	7.757	7.124	-11.63	-82.96
CPD5	7.227	7.340	7.210	6.572	-12.24	-82.15
CPD6	7.093	6.953	7.691	7.207	-10.43	-80.95
CPD7	7.628	7.651	7.567	7.160	-11.00	-80.74
CPD8	7.070	6.896	7.573	7.077	-10.33	-80.31

CPD9	7.165	7.227	7.102	7.076	-10.71	-79.65
CPD10	7.047	7.192	7.027	7.240	-11.04	-79.63
CPD11	7.206	7.002	7.030	6.596	-11.11	-77.50
CPD12	7.092	7.006	7.165	6.539	-10.32	-77.11

Table S6. Chemical similarity between MELK hits and their closest ligands.

Compound ID	MELK hit	Closest ligand (MELK inhibitors)	ECFP Similarity
CPD1	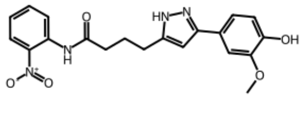	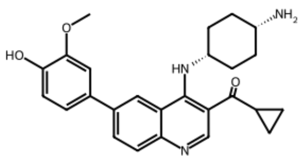	0.253
CPD2	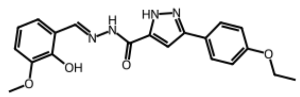	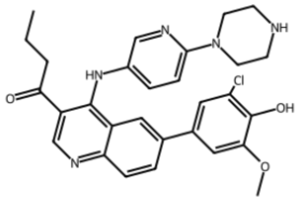	0.245
CPD3	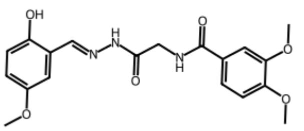	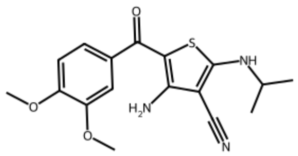	0.297
CPD4	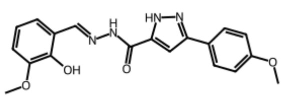	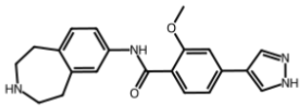	0.244

CPD5	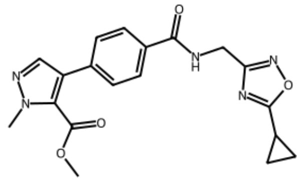	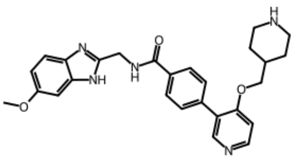	0.305
CPD6	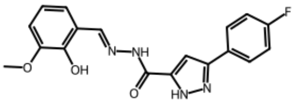	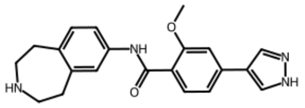	0.244
CPD7	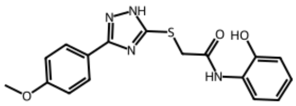	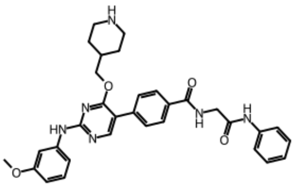	0.25
CPD8	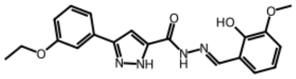	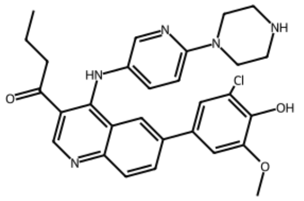	0.231
CPD9	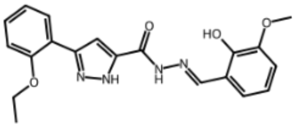	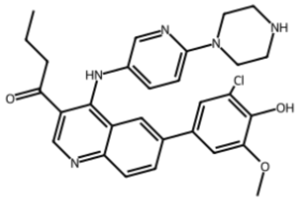	0.235

CPD10	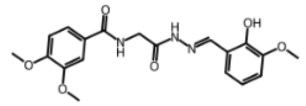	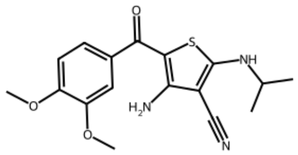	0.284
CPD11	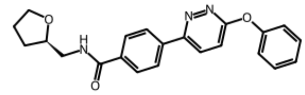	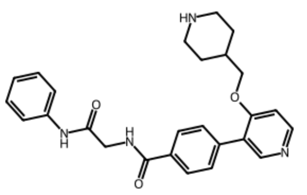	0.279
CPD12	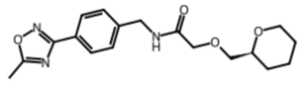	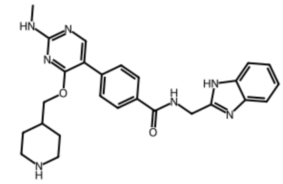	0.242
